# Late weaning and maternal closeness, associated with advanced motor and visual maturation, reinforce autonomy in healthy, 2-year-old children

**DOI:** 10.1038/s41598-020-61917-z

**Published:** 2020-03-23

**Authors:** José Villar, Roseline Ochieng, Eleonora Staines-Urias, Michelle Fernandes, Marc Ratcliff, Manorama Purwar, Fernando Barros, Bernardo Horta, Leila Cheikh Ismail, Elaine Albernaz, Naina Kunnawar, Sophie Temple, Francesca Giuliani, Tamsin Sandells, Maria Carvalho, Eric Ohuma, Yasmin Jaffer, J. Alison Noble, Michael Gravett, Ruyan Pang, Ann Lambert, Enrico Bertino, Paola Di Nicola, Aris Papageorghiou, Alan Stein, Zulfiqar Bhutta, Stephen Kennedy

**Affiliations:** 1 Nuffield Department of Women’s & Reproductive Health, University of Oxford and Oxford Maternal & Perinatal Health Institute, Green Templeton College, University of Oxford, Oxford, UK; 2 Faculty of Health Sciences, Aga Khan University, Nairobi, Kenya; 3 Nuffield Department of Women’s & Reproductive Health, University of Oxford, Oxford, UK; 4 Faculty of Psychology and Educational Science, University of Geneva, Geneva, Switzerland; 5 Nagpur INTERGROWTH-21st Research Centre, Ketkar Hospital, Nagpur, India; 6 Programa de Pós-Graduação em Saúde e Comportamento, Universidade Católica de Pelotas, Pelotas, Brazil; 7 Postgraduate Program in Epidemiology, Universidade Federal de Pelotas, Pelotas, Brazil; 8 College of Health Sciences, University of Sharjah, Sharjah, United Arab Emirates; 9 Faculty of Medicine, Universidade Federal de Pelotas, Pelotas, Brazil; 10 Ospedale Infantile Regina Margherita-Sant’Anna Citta della Salute e della Scienza di Torino, Torino, Italy; 11 Centre for Statistics in Medicine, Nuffield Department of Orthopaedics, Rheumatology & Musculoskeletal Sciences, University of Oxford, Oxford, UK; 12 Department of Family & Community Health, Ministry of Health, Muscat, Oman; 13 Department of Engineering Science, University of Oxford, Oxford, UK; 14 Departments of Obstetrics and Gynecology and of Global Health, University of Washington, Seattle, USA; 15 School of Public Health, Peking University, Beijing, China; 16 Dipartimento di Scienze Pediatriche e dell’ Adolescenza, SCDU Neonatologia, Universita di Torino, Torino, Italy; 17 Department of Psychiatry, Warneford Hospital, University of Oxford, Oxford, UK; 18 Center for Global Child Health, Hospital for Sick Children, Toronto, Canada

**Keywords:** Medical research, Paediatric research

## Abstract

We studied neurodevelopmental outcomes and behaviours in healthy 2-year old children (N = 1306) from Brazil, India, Italy, Kenya and the UK participating in the INTERGROWTH-21^st^ Project. There was a positive independent relationship of duration of exclusive breastfeeding (EBF) and age at weaning with gross motor development, vision and autonomic physical activities, most evident if children were exclusively breastfed for ≥7 months or weaned at ≥7 months. There was no association with cognition, language or behaviour. Children exclusively breastfed from birth to <5 months or weaned at >6 months had, in a dose-effect pattern, adjusting for confounding factors, higher scores for “emotional reactivity”. The positive effect of EBF and age at weaning on gross motor, running and climbing scores was strongest among children with the highest scores in maternal closeness proxy indicators. EBF, late weaning and maternal closeness, associated with advanced motor and vision maturation, independently influence autonomous behaviours in healthy children.

## Introduction

The relationship between infants and their mothers is of paramount importance to child development, and ultimately for the survival of the species^1^. Humans are born immature, from a developmental perspective, and are totally dependent for the allostatic regulation of their physiological processes^2^, requiring more prolonged supported feeding than other mammals. During this period of newborn brain plasticity and vulnerability, early human development can be markedly influenced by environmental (including nutritional) factors^3^.

Consequently, the mother-infant dyad, as it relates to breastfeeding, has been the subject of studies in philosophy, psychoanalysis, developmental psychology, biology, sociology and economics, and more recently neuroscience, epigenetics and immunology. Considerable evidence exists to support the health, nutritional and economic benefits of breast milk up to adulthood^4–6^; the importance of “bonding” and “attachment” (i.e. direct contact between the infant and the breast, nipple and maternal skin)^7^, and the visual and olfactory interactions that accompany the act of breastfeeding^8,9^.

These complex interpersonal processes have been subject to numerous interpretations, in part because the mechanisms achieving such effects are uncertain. Nevertheless, the sources agree that the duration of intimate contact between mother and infant, and the subsequent separation processes, are critical components of human neurodevelopment. They manifest more evidently when the child confronts its wider social environment, usually by 2 years of age.

Studying these processes whilst controlling for so many different factors is challenging. To our knowledge, there has been no attempt to study these developmental processes prospectively in the context of a large, healthy cohort of mothers and their infants, enrolled early in pregnancy and living in a variety of modern societies, under environmental influences that are relatively stable.

A prospective study, designed to explore the relationship between breastfeeding/weaning and the different stages of neurodevelopment, would be a unique psycho-biological model, which might provide insights into the mechanisms proposed as the early drivers of cognition, as well as mental wellbeing and disease. We contend that our recent study of a cohort of well-nourished children, living under favourable health, environmental and nutritional conditions, whose growth and development have been monitored from early fetal life (The INTERGROWTH-21^st^ Project), provides such an opportunity^10^.

We first demonstrated in this cohort that the variability in neurodevelopment and related behaviours at 2 years of age is considerably smaller between geographically diverse populations than within individual populations^11,12^. We explore here the association between the timing/duration of breastfeeding and age at weaning, and domain-specific neurodevelopmental skills and behaviours at 2 years of age.

## Results

**Figure 1:**
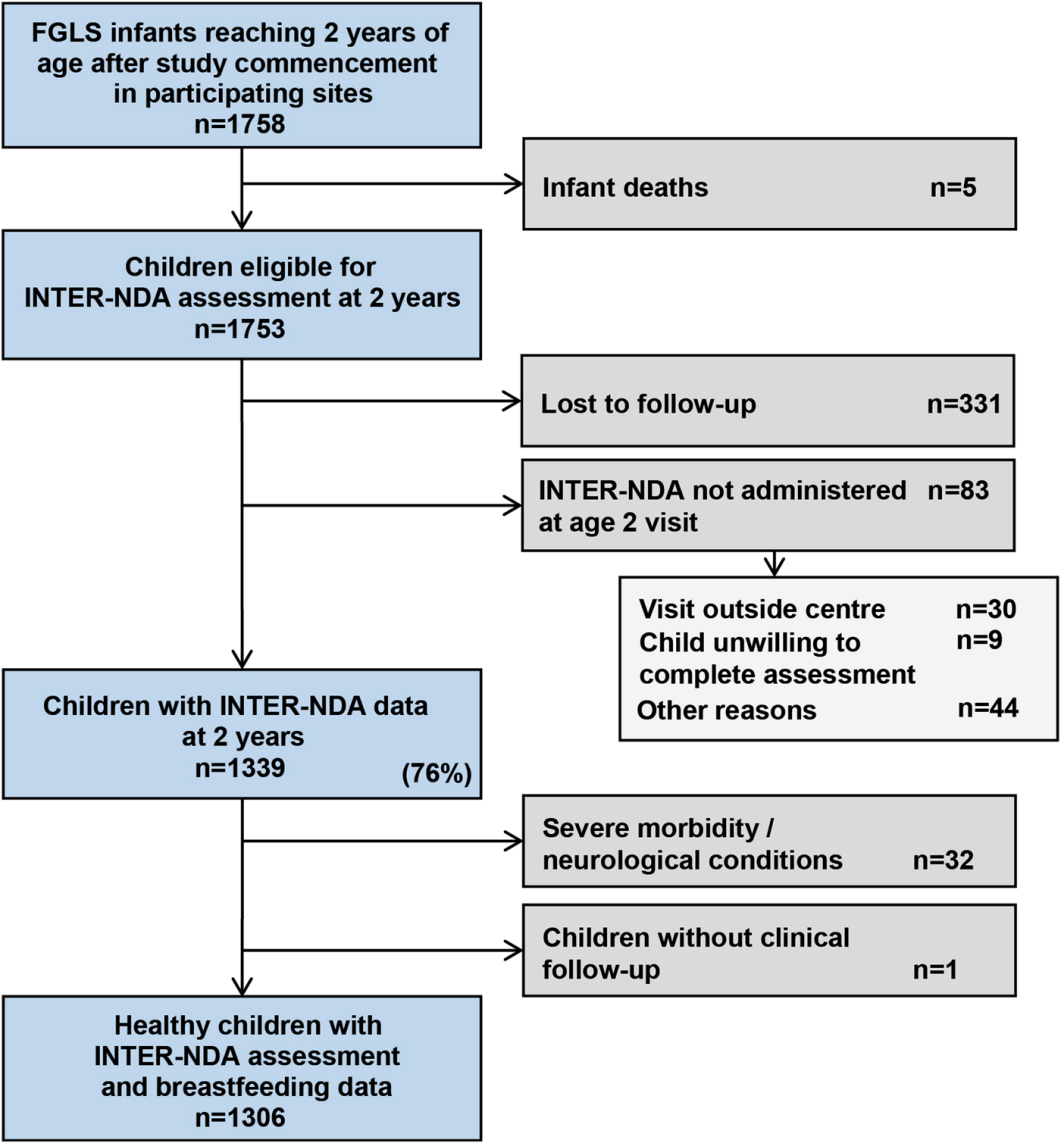
INTERGROWTH-21^st^ Project Infant Follow-up Study: participants’ flow. FGLS = Fetal Growth Longitudinal Study. NDA = Neurodevelopment Assessment. Exclusions: meningitis, hearing loss, blindness or major visual problems, seizures, cerebral palsy, neurological disorders (such as epilepsy), malignancy, malaria, tuberculosis, hepatitis, HIV/AIDS, cystic fibrosis or haemolytic conditions diagnosed at the 1- or 2-year visits.

There were 1753 children enrolled in the INTERGROWTH-21^st^ Fetal Growth Longitudinal Study (FGLS) that were alive at 2 years of age within the study’s data collection period. Among these, 1339 (76.4%) completed the neurodevelopmental assessment close to their second birthday. However, 32 children were excluded from this analysis because of a diagnosis of severe visual problems, seizures, hearing impairment, malaria or a heart condition made or reported at the time of the 2-year visit; another child was excluded because, despite having completed the developmental evaluation, feeding data at 1 year of age were not available. Thus, we present detailed neurodevelopmental information from 1306 children (682 girls and 624 boys) (Fig. 1).

Each site’s proportional contribution was 15% Brazil (N = 198), 24% India (N = 318), 24% Italy (N = 311), 24% Kenya (N = 311) and 13% the UK (N = 168). The mean age of the children at the time of assessment was 25.05 months (SD 2.15 months); median 24.25 months; (IQR 23.92, 25.17 months).

To take into consideration fetal growth patterns that might directly have influenced the neurodevelopmental scores of these children, we used the ultrasound measures of their head circumference prospectively obtained in FGLS between 25 and 30 weeks’ gestation. The centile distributions of fetal head circumference of the studied sample were almost identical to the INTERGROWTH-21^st^ Fetal Growth Standards^13^. The mean z-score of the sample was 0.00 (SD 0.98 for this gestational age range), i.e. there was no evidence that any of the fetal heads in the study sample were growth restricted (Supplementary Fig. 1).

Similar birth characteristics and early neonatal morbidity rates were found in the sample studied (N = 1306), the children lost to follow-up (N = 331) (Table 1), and the full cohort^11^ demonstrating that the study children could be considered a representative sample of the FGLS population. Furthermore, anthropometric measures at 2 years of age, assessed against the World Health Organization (WHO) Child Growth Standards^14^, were compatible with a well-nourished population. Morbidity episodes reported at the 2-year follow-up visit were very similar to those of the full cohort of healthy infants included in FGLS^11^; in other words, the children had very low rates of clinical conditions that could have led to sub-optimal size, morbidity and/or neurodevelopment delay at 2 years of age (Table 2).

**Table 1:** Neonatal characteristics of children completing the INTERGROWTH-21^st^ Neurodevelopment Assessment (INTER-NDA) in the Infant Follow-up Study of the INTERGROWTH-21^st^ Project compared to children lost to follow-up.

	Children evaluated (n = 1306)	Children lost to follow-up (n = 331)
Gestational age at delivery, weeks	39.4 (1.5)	39.3 (1.5)
Birthweight, kg	3.2 (0.5)	3.2 (0.5)
Birth length, cm	49.1 (2.0)	49.0 (2.1)
Head circumference at birth, cm	33.9 (1.4)	34.0 (1.3)
Apgar at 5 min	9.5 (0.6)	9.6 (0.7)
Age at hospital discharge, days^a^	3.0 (2.0, 4.0)	2.0 (1.0, 3.0)
Boys	624 (47.8)	160 (48.3)
Preterm birth (<37 weeks’ gestation)	54 (4.1)	16 (4.8)
Early preterm (<34 weeks’ gestation)	6 (0.5)	2 (0.6)
NICU stay>1 day; <3 days	47 (3.6)	18 (5.5)
NICU > 3 days	33 (2.5)	12 (3.6)
Hyperbilirubinaemia	66 (5.1)	18 (5.5)
Respiratory distress syndrome	29 (2.2)	7 (2.1)
Transient tachypnoea of the newborn	18 (1.4)	12 (3.6)
Exclusive/predominant breastfeeding at hospital discharge	1249 (95.6)	305 (92.1)

Data are mean (SD) or proportion (%) unless otherwise specified. Missing data less than 2% for all variables.

^a^ Median (inter-quartile range).

NICU: neonatal intensive care unit.

**Table 2:** Anthropometric measures and morbidity by 2 years of age of children who completed the INTERGROWTH-21^st^ Neurodevelopment Assessment (INTER-NDA) in the Infant Follow-up Study of the INTERGROWTH-21^st^ Project (n = 1301^a^).

Anthropometric measure or index	Mean (SD)
Weight, kg	12.3 (1.6)
Length, cm	86.8 (3.4)
Body mass index, kg/m^2^	16.4 (1.7)
Head circumference, cm	47.9 (1.6)
Weight z-score	0.2 (1.1)
Length z-score	−0.2 (1.0)
Body mass index z-score	0.4 (1.2)
Head circumference z-score	0.1 (1.1)
**Medical condition**	**N (%)**^**b**^
Hospitalised at least once	116 (8.9)
Total number of days hospitalised (median, IQR)	2 (1, 4)
Any prescription made by a health care professional	768 (59.0)
Antibiotics (≥3 regimens)	163 (12.5)
Iron/folic acid/vitamin B12/other vitamins	215 (16.5)
Up-to-date with local vaccination policies	1226 (94.2)
Otitis media/Pneumonia/Bronchiolitis	97 (7.5)
Parasitosis/Diarrhoea/Vomiting	51 (3.9)
Exanthema/skin disease	159 (12.2)
UTI/pyelonephritis	6 (0.5)
Fever ≥3 days (≥3 episodes)	146 (11.2)
Other infections requiring antibiotics	44 (3.4)
Asthma	15 (1.2)
Gastro-oesophageal reflux	4 (0.3)
Cow’s milk protein allergy	11 (0.8)
Food allergies	15 (1.2)
Injury trauma	34 (2.6)
Any condition requiring surgery	9 (0.7)

Age- and sex-specific z-scores compared to the World Health Organization Child Growth Standards^14^, using corrected age for infants born preterm.

^a^Clinical follow-up data at 2 years not available for 5 (0.4%) children. Missing data less than 2% for all variables.

^b^N (%) except for hospitalisation days for which median (interquartile range) are presented.

Ninety-six percent of the newborns were being exclusively/predominantly breastfed at hospital discharge (Table 1). They continued to be exposed to a high level of exclusive/predominant breastfeeding practices: 32% up to the first 5 postnatal months, 58% from birth up to less than 7 months, and 4% from birth to 7 months or more. In addition, 56% were exposed to any form of breastfeeding for more than 13 months, 23% for more than 7 months but less than 13 months, 8% for more than 5 months but less than 7 months, and only 12% were exposed to less than 5 months of any breastfeeding during the first 2 years of life. Only 23 infants (1.8%) were never breastfed.

These feeding patterns are the consequence of the strong support and advice about breastfeeding practices provided to these well-educated mothers throughout pregnancy and the neonatal period by the dedicated research staff involved in the INTERGROWTH-21^st^ Project. The attending health care professionals and research staff also provided constant reinforcement during infancy of the advantages of breast milk and feeding strategies to support extended breastfeeding.

Among the 761 infants that were still being exposed to any breastfeeding up to 1 year of age, 31.1% had between 1–3 feeds per day, 46.7% had 4–6 feeds per day and 22.2% had more than 7 feeds per day. Infants who were not having any breast milk at 1 year of age (N = 407) were taken as the “baseline category” in the association analysis.

Mothers reported that 597 (47.6%) children at 2 years of age had never been given formula feeds. Of the remaining children, 232 (18.5%) were exposed to formula feeding for a period of less than 5 months; 74 (5.9%) had formula for a period longer than 5 months but less than 7 months; 229 (18.3%) for 7 to less than 13 months, and 122 (9.7%) were fed with formula for more than 13 months. For the 661 infants who had formula feeds, the mean age at the time of introducing formula was 4.6 (SD 3.9) months, with a median of 4.2 months (IQR 1.5, 6.0 months). Hence, given the variability in the periods during which formula was used, in all analyses we have adjusted by the infant’s chronological age at the initiation of formula use and by the age periods during which they were formula fed.

**Figure 2:**
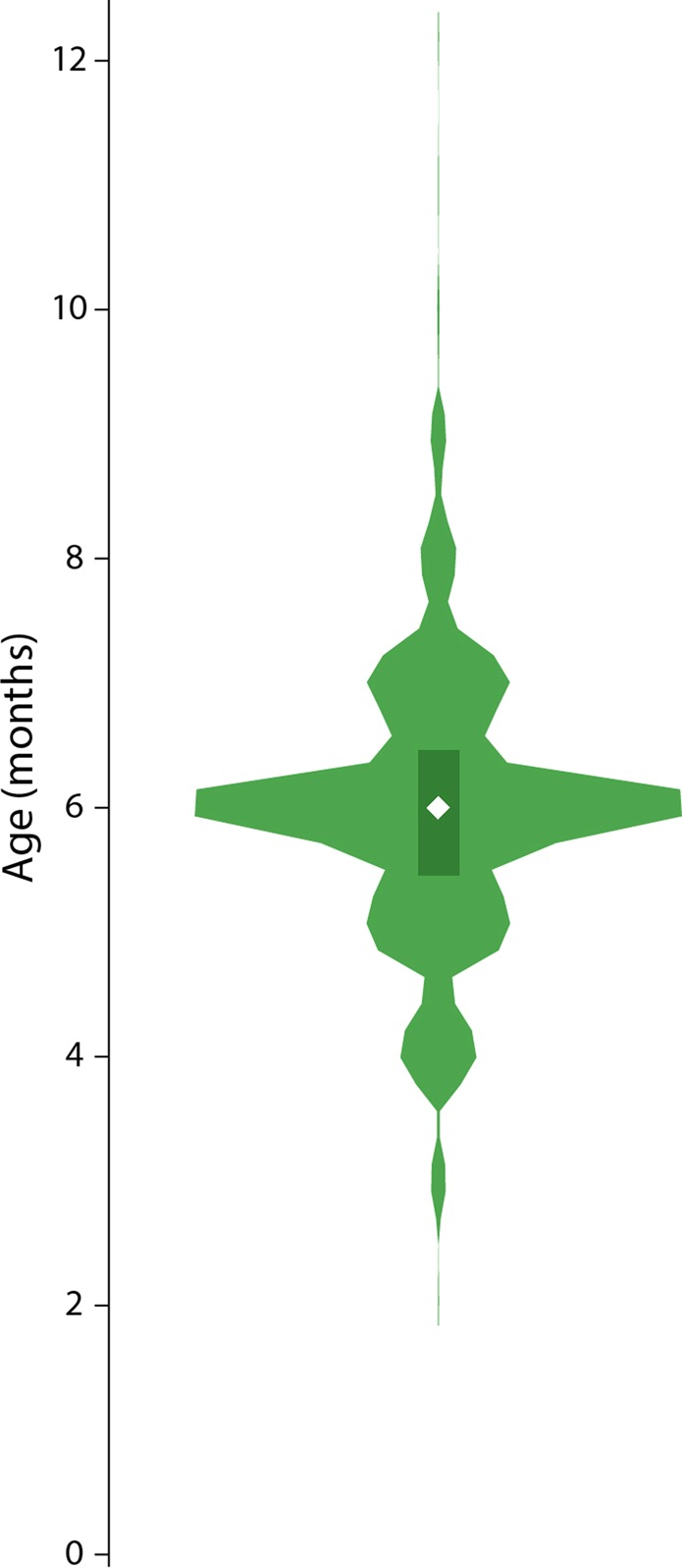
“Violin” plot showing the distribution of age at weaning for children included in the INTERGROWTH-21^st^ Neurodevelopment Assessment Study (N = 1260)*. The vertical axis represents the age (in months) at which solids started in the study sample. The median weaning age (white diamond) was 6 months (IQR 5.5–7.5 months; dark green box). *46 children (3.5%) had no data on age at weaning.

We have treated the chronological age at weaning (as a continuous and categorical variable) as one of the two main “exposure” variables. The age distribution of weaning is presented in Fig. 2 in the format of a “violin” plot. As can be seen, the median age at weaning was 6 months (IQR 5.5–6.5) with the distribution having the largest peak at the median (6 months) and smaller peaks at 5 and 7 months. There were 387 infants exposed to solid/semi-solid foods before 6 months; 463 during the 6^th^ month; 181 during the 7^th^ month, and 229 after 7 months of age.

Feeding practices during infancy have been consistently reported as associated with morbidity episodes, in particular infection/allergic-related conditions in a variety of populations worldwide^15,16^. Hence, we explored the association in our healthy, low-risk cohort, living in adequate environmental conditions. By 2 years of age, 173 (13.3%) children overall had had 1–2 episodes of a moderate clinical morbidity diagnosis, as described above (children with severe diseases were excluded from the sample) and 354 (27.2%) had had 1–2 episodes of infection-related morbidity that required a medical consultation and/or treatment.

The association between the five feeding variables and the rate of infection-related morbidity by 2 years of age (adjusted OR; 95% CI) was estimated. There was a consistent protective effect of exclusive breastfeeding for infection-related morbidity rates, especially for infants who were exclusively breastfed from birth to 5–7 months compared with those without exclusive breastfeeding, after adjusting for the variables identified as possible confounders (Supplementary Fig. 2).

Similarly, delaying weaning after 6 months (compared with those starting weaning less than 6 months) led to a lower risk of infection-related morbidity, again after adjusting for the possible confounding factors. Conversely, duration of formula use, adjusted by the timing of formula use, even in these low-risk environments, was associated with a consistent trend towards increased risk (Supplementary Fig. 2).

We also explored the relationship between the indicators of breastfeeding, weaning and formula feeding and body mass index (BMI) at 2 years of age, based on the WHO Child Growth Standards^14^. After adjusting for age at the time of INTER-NDA testing, sex, gestational age at birth, birth weight, birth length, Neonatal Intensive Care Unit (NICU) stay, maternal education and age, fetal head circumference z-score and postnatal smoking exposure (here, we did not adjust for height at 2 years of age), we found a systematic pattern. There was a negative relationship between BMI and duration of exclusive/predominant breastfeeding in months (−0.049; −0.091, −0.008), number of breastfeeds at 1 year of age (−0.074; −0.102, −0.045), duration of any breastfeeding (−0.013; −0.024, −0.001) and age at introduction of solid/semi-solid foods (−0.085; −0.166, −0.004), i.e. the longer the breastfeeding exposure and the later the age at weaning, the lower the BMI at 2 years of age.

In short, the analyses presented above are in agreement with the extensive literature on the preventive effect of breastfeeding on early childhood morbidity, associated with lower BMI and overweight prevalence^17,18^. The next set of analyses, the primary aims of this paper, describe the relationship between feeding practices during infancy/early childhood and a set of neurodevelopmental domains and related behaviours^12^.

### Association between feeding practices and neurodevelopmental domains

**Figure 3:**
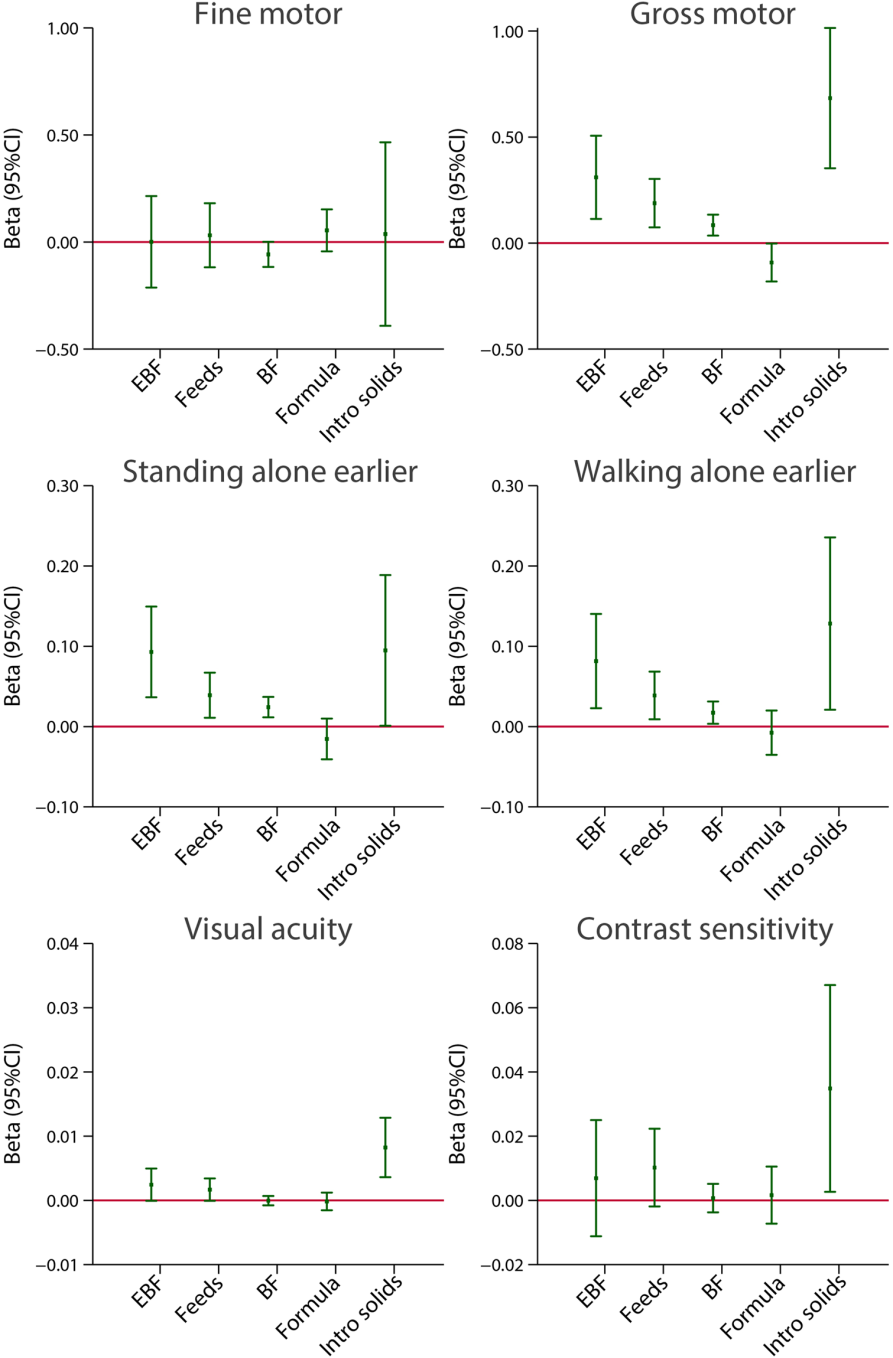
Association between breastfeeding exposures and motor and visual neurodevelopmental domains at 2 years of age. The central dots represent the change in the neurodevelopmental score (with vertical lines representing 95% CIs) for each unit increase in the continuous exposure. Results from linear regression models adjusted for age at assessment, sex, gestational age at birth, birth weight, birth length, NICU stay, maternal education, maternal age, fetal head circumference z-score, postnatal smoking exposure, and length z-score at 2 years. For formula use, we further adjusted by the timing of initiation. With robust standard errors. EBF = duration of exclusive breastfeeding in months. Feeds = number of breast milk feeds per day. BF = duration of any breastfeeding in months. Formula = duration of formula use in months. Intro solids = weaning age in months.

**Figure 4:**
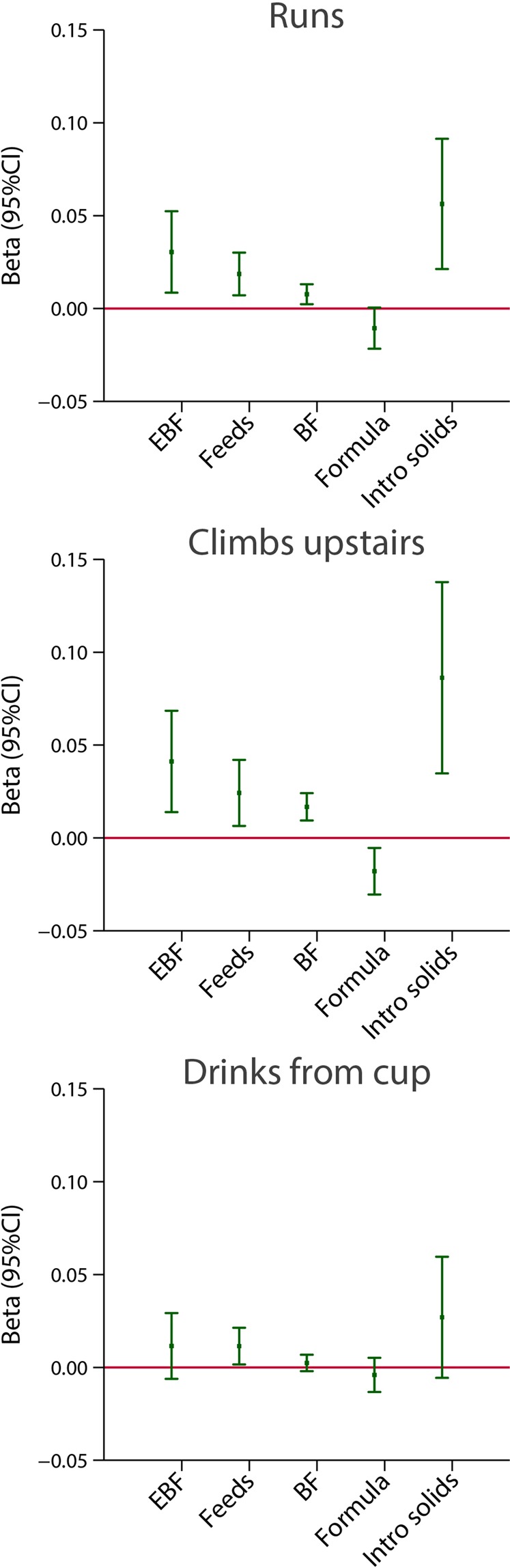
Association between breastfeeding exposures and INTER-NDA markers of child autonomy at 2 years of age. The central dots represent the change in the neurodevelopmental score (with vertical lines representing 95% CIs) for one unit increase in the continuous exposure. Results from linear regression models adjusted for age at assessment, sex, gestational age at birth, birth weight, birth length, NICU stay, maternal education, maternal age, fetal head circumference z-score, postnatal smoking exposure, and length z-score at 2 years. For formula use, we further adjusted by the timing of initiation. With robust standard errors. EBF = duration of exclusive breastfeeding in months. Feeds = number of breast milk feeds per day. BF = duration of any breastfeeding in months. Formula = duration of formula use in months. Intro solids = weaning age in months.

Figure 3 presents the adjusted linear regression coefficients (95% CI) between the five indicators of feeding practices as continuous variables and the scores of fine and gross motor domains, as well as visual tests also expressed as continuous variables. It illustrates that, except for fine motor, there is a consistent pattern towards higher domain-specific scores of gross motor, age at the earliest standing alone and walking alone and visual acuity according to increasing levels of breastfeeding exposure, i.e. the greater the number of months of exclusive/predominant breastfeeding, number of breastfeeds per day and total months exposed to any breastfeeding, the higher the scores of the above domains or items. Similar effects are presented in Fig. 4 for runs, climbs upstairs without assistance and drinks from a cup spontaneously, three markers of the child’s autonomy from the mother.

A consistent effect was seen between the age at weaning (the later the weaning) and the scores (the higher the score) across these eight items. The effect was strongest for the INTER-NDA gross motor, WHO milestones runs and climbs upstairs, and Cardiff visual score (Figs. 3 and 4).

A mirror trend in the effect was observed when the analysis was performed using total duration of formula use (in months), adjusted for the age on initiation of formula use, as the independent variable: longer duration (months) of formula use was systematically associated with lower scores in five of the eight neurodevelopmental domains/items after adjusting for the set of confounding variables. Such an association was not observed with the fine motor, early walking and vision outcome (Figs. 3 and 4).

**Figure 5:**
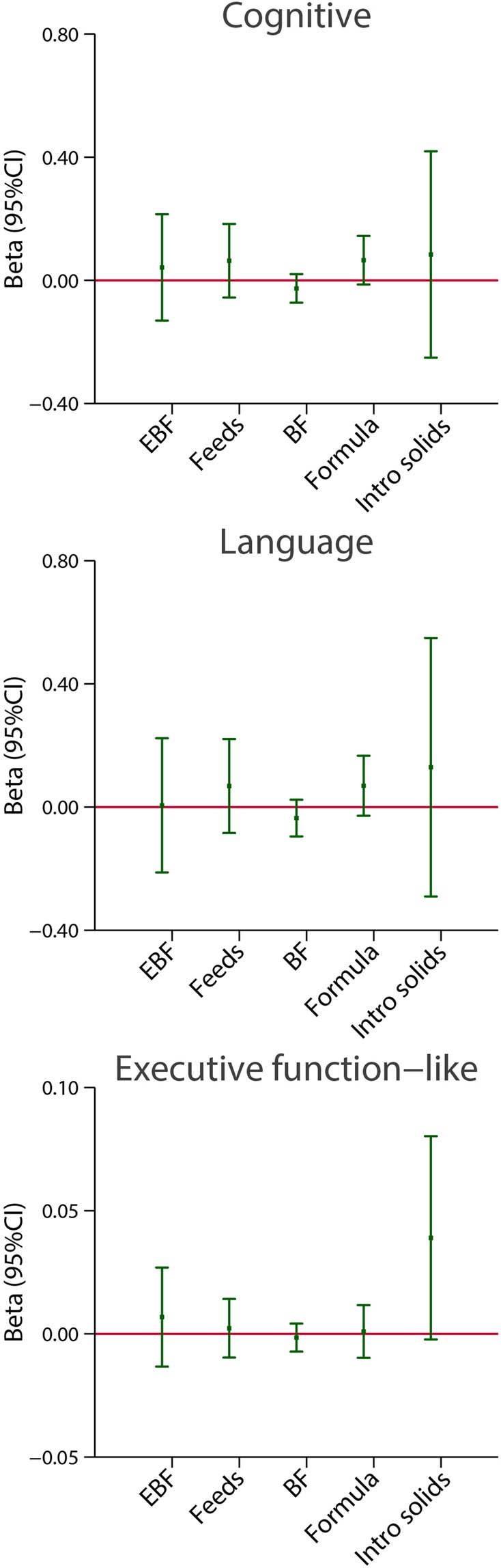
Association between breastfeeding exposures and cognitive and language domains, as well as the “executive function” item at 2 years of age. The central dots represent the change in the neurodevelopmental score (with vertical lines representing 95% CIs) for each unit increase in the continuous exposure. Results from linear regression models adjusted for age at assessment, sex, gestational age at birth, birth weight, birth length, NICU stay, maternal education, maternal age, fetal head circumference z-score, postnatal smoking exposure, and length z-score at 2 years. For formula use, we further adjusted by the timing of initiation. With robust standard errors. EBF = duration of exclusive breastfeeding in months. Feeds = number of breast milk feeds per day. BF = duration of any breastfeeding in months. Formula = duration of formula use in months. Intro solids = weaning age in months.

Figure 5 presents results, using the same analytical strategy, for the same five markers of feeding practices as independent variables, but focused here on the cognitive, language and an “executive function-like” item as the dependent variables, all expressed as continuous scores and presented as linear regression coefficients (95% CI) adjusted for the same variables as above. There were no clear patterns of association in these domains except for a trend in the “executive function-like” item’s score where there was a positive association with the age at weaning (the later weaning occurred, the higher the score), although with a wide adjusted 95% CI (Fig. 5).

**Figure 6:**
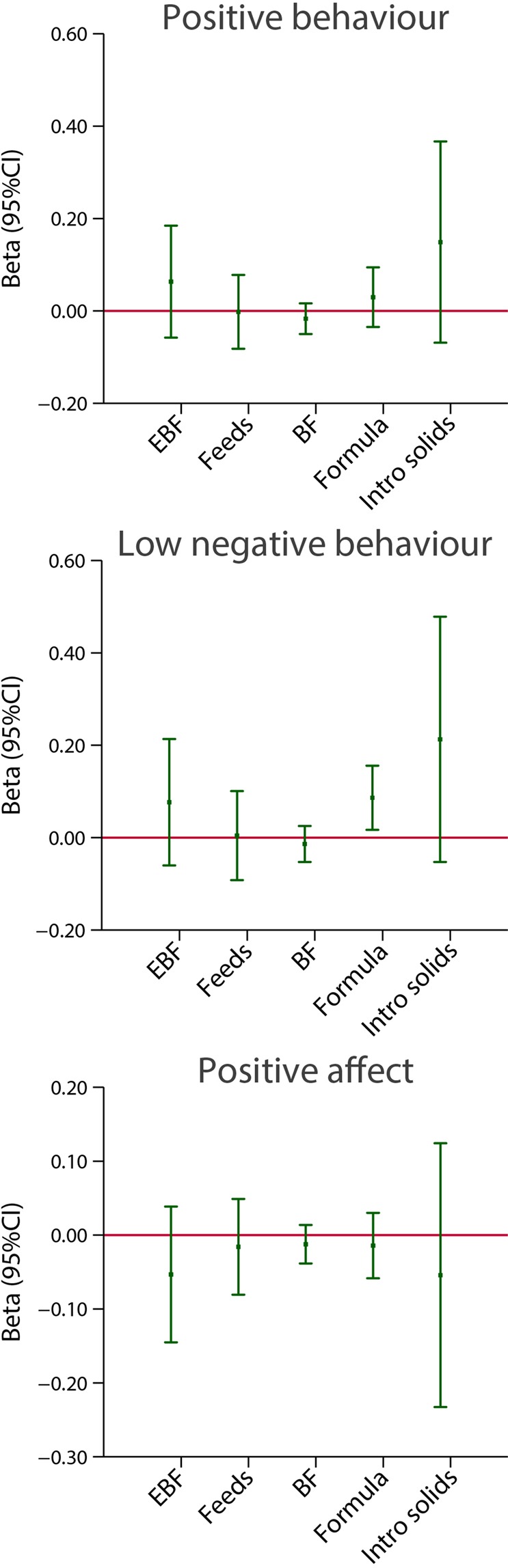
Association between breastfeeding exposures and three behavioural domains at 2 years of age. The central dots represent the change in the neurodevelopmental score (with vertical lines representing 95% CIs) for each unit increase in the continuous exposure. Results from linear regression models adjusted for age at assessment, sex, gestational age at birth, birth weight, birth length, NICU stay, maternal education, maternal age, fetal head circumference z-score, postnatal smoking exposure, and length z-score at 2 years except for BMI at 2 years. For formula use, we further adjusted by the timing of initiation and periods covered. With robust standard errors. EBF = duration of exclusive breastfeeding in months. Feeds = number of breast milk feeds per day. BF = duration of any breastfeeding in months. Formula = duration of formula use in months. Intro solids = weaning age in months.

Positive and low negative behaviour, as well as the positive affect domain, the latter including items such as “like playing with other children” or “responds well to affections”, were similarly evaluated in their association with the five feeding practice indicators. There was no consistent association or trend between feeding practices and these neurodevelopmental domains (Fig. 6).

The attentional “problems” and “emotional reactivity” domains from the Child Behavior Checklist (CBCL) scale^19^ based on the mother’s report of the child’s behaviour, were evaluated. Very high scores in these domains are often used to identify behavioural problems in children from 18 months to 5 years of age^20^. Given the low-risk profile of our sample, we hypothesised *a priori* that these children would be very unlikely to have scores in the high-risk (clinical) range of the scale. The descriptive data confirmed this prediction: for example, the range of possible values for “emotional reactivity” score on this scale, as implemented in the study, was 0 to 20. In our sample, the mean and median scores were 3.9 (SD 3.1) and 3.3 (IQR 2.2,5.6), respectively; 95% of the children had scores less than 9.

For the attentional “problems” score, the range of possible values on the CBCL scale, as implemented by us, was also 0 to 20. In our sample, the mean was 5.7 (SD 4.0) and the median 6.0 (IQR 2.0,8.0); 95% scored less than 12. Therefore, the reported scores reflect values within the normal range of the scale.

**Figure 7:**
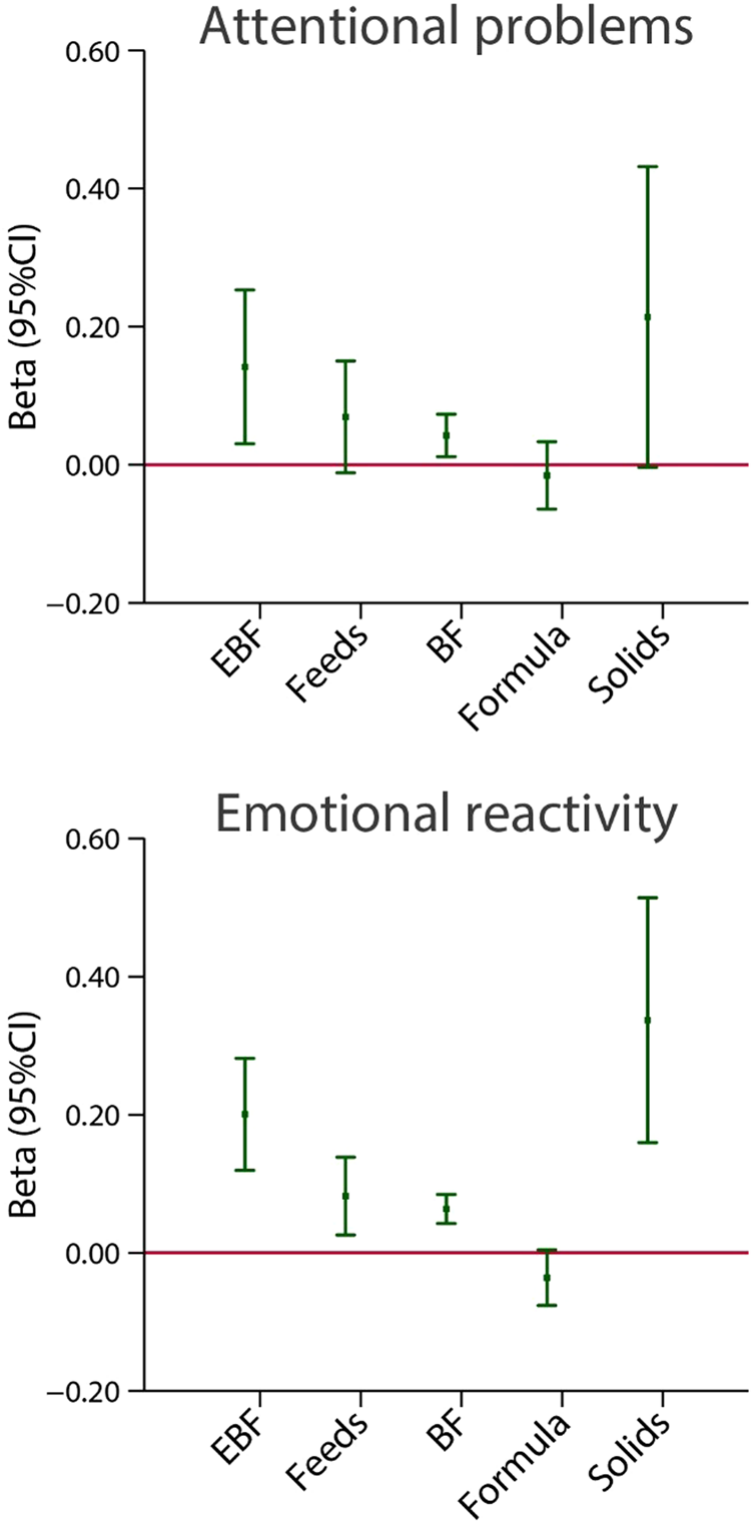
Association between breastfeeding exposures and attentional “problems” and “emotional reactivity” domains scores at 2 years of age. Attentional “problems” reflects the child’s scores on the attentional problems subscale of the preschool version of the Child Behavior Checklist (CBCL). In this sample, none of the children scored above the clinical or borderline clinical thresholds for attentional problems on the CBCL; therefore, the scores reflect the variation within the normal range. The central dots represent the change in the neurodevelopmental score (with vertical lines representing 95% CIs) for each unit increase in the continuous exposure. Results from linear regression models adjusted for age at assessment, sex, gestational age at birth, birth weight, birth length, NICU stay, maternal education, maternal age, fetal head circumference z-score, postnatal smoking exposure, and length z-score at 2 years. For formula use, we further adjusted by the timing of initiation. With robust standard errors. EBF = duration of exclusive breastfeeding in months. Feeds = number of breast milk feeds per day. BF = duration of any breastfeeding in months. Formula = duration of formula use in months. Intro solids = weaning age in months.

Very similar patterns to those shown in Figs. 3 and 4 for motor and visual domains emerge for the association between scores from the attentional “problems” scale and “emotional reactivity” and breastfeeding indicators (Fig. 7), i.e. the greater the breastfeeding exposure (exclusive/predominant breastfeeding; number of breastfeeds and duration of any breastfeeding exposure) as well as the older the child at the age of weaning, the higher the attentional “problems” scores (within normal values) and “emotional reactivity” scores (Fig. 7).

We also compared the individual raw scores from our sample against the recommended CBCL scale, using the web-based program provided by the CBCL/1.5–5 syndrome scale scores^21^, to identify so-called “clinical cases”, i.e. greater than 97^th^ centile of the CBCL scale. Only 24 (1.8%) and 12 (0.9%) children in our sample on the attentional and “emotional reactivity” domains respectively, could be considered clinical cases; seven of these children had clinical values in both domains. Therefore, we conducted sensitivity analyses for these two developmental outcomes excluding these 29 children from the study sample.

The pattern of the associations described above were identical with those excluded, with only slightly wider confidence intervals in the attentional domain for any breastfeeding and introduction of solids. Hence, our results should be interpreted as variations within the normal levels of these scales, i.e. the scores are those of active, independent or interested children, rather than those of children with attentional “problems” or hyperactivity.

To improve our understanding of these results, we explored whether the attentional “problems” scores were related to scores that are indicators of motor delay; no such association was observed. Similarly, we explored the association between items collected by direct observation of the child at the time of the assessment that were scored as “unable to assess” because the child had difficulty co-operating with the assessor or the child was too distracted to complete tasks; versus the attentional “problems” and “emotional reactivity” reported by the mother on the CBCL. This analysis aimed at comparing the mother’s perception of the child’s behaviour at home with that observed in the assessment clinic. No pattern emerged between higher attentional “problems” scores on the CBCL and the rate of “unable to assess” on the INTER-NDA (Spearman correlation coefficient = −0.17). Finally, we considered in all the multivariable analyses, height at 2 years of age as a possible nutritional mediator of the observed associations. Excluding this variable from the regression models did not modify in any substantive or systematic way the associations presented in the figures.

### Association between duration of exclusive/predominant breastfeeding and age at weaning and neurodevelopmental domains according to the timing of feeding exposure

In this second set of analyses, with a focus on the two feeding indicators (exclusive/predominant breastfeeding and age at weaning), we explored whether there was an age window(s) of the effect or “dose-dependent effect” pattern that was consistently associated with specific neurodevelopmental domains/items at 2 years of age. We stratified the duration of exclusive/predominant breastfeeding by age brackets from birth to less than 5 months (N = 403), from birth to greater than 5 months but less than 7 months (N = 731), and from birth to 7 months or more but less than 13 months (N = 53). We used children with no exclusive/predominant breastfeeding (N = 67) as the baseline group (i.e. children discharged from hospital after birth on any feeding combination other than exclusive breastfeeding).

We conducted the same analysis for categories of the age at which solid/semi-solid foods were introduced, i.e. the “weaning effect”: solid/semi-solid foods introduced less than 6 months as the baseline group (N = 387), between 6 and 7 months (N = 644) and at/or after 7 months of age (N = 229). All results were adjusted by the same variables as in the previous analyses. We attempted to identify a specific timing effect or window of sensitivity for the overall associations observed in the continuous analyses presented in Figs. 3 to 7.

**Figure 8:**
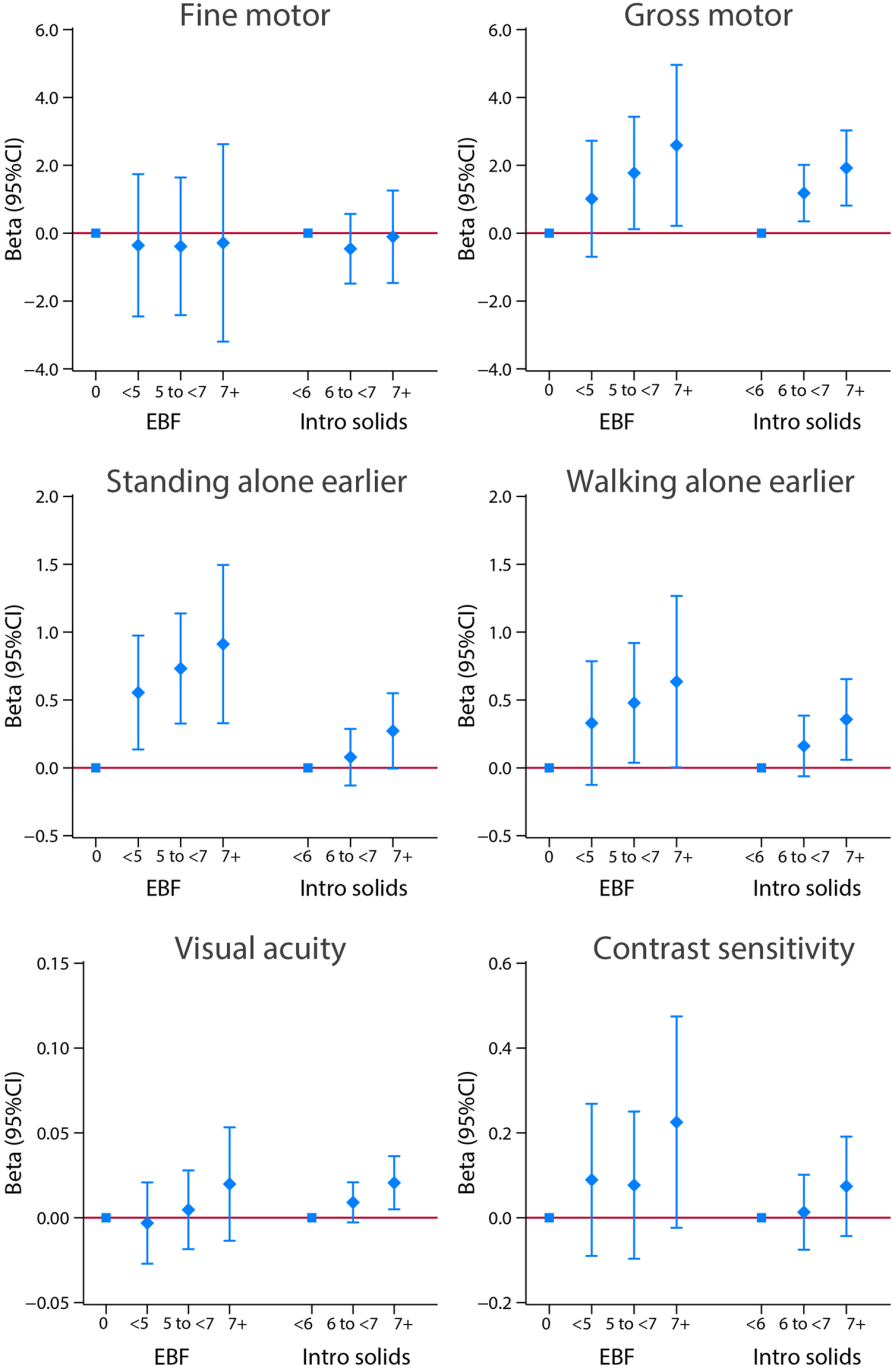
Association between categories of length of exclusive breastfeeding (EBF) and weaning age (introduction of solids), in months, and motor and vision outcomes at 2 years of age. Results from linear regression models adjusted for age at assessment, sex, gestational age at birth, birth weight, birth length, NICU stay, maternal education, maternal age, fetal head circumference z-score, postnatal smoking exposure, and length z-score at 2 years. With robust standard errors.

**Figure 9:**
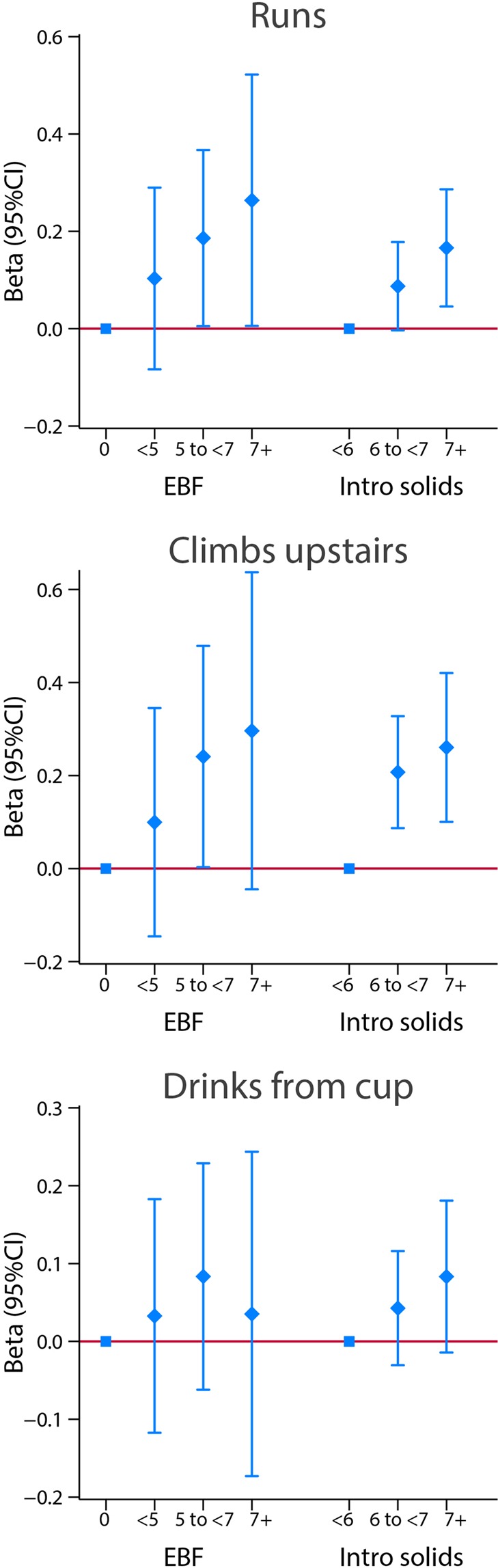
Association between categories of length of exclusive breastfeeding (EBF) and weaning age (introduction of solids), in months, and three INTER-NDA markers of child autonomy at 2 years of age. Results from linear regression models adjusted for age at assessment, sex, gestational age at birth, birth weight, birth length, NICU stay, maternal education, maternal age, fetal head circumference z-score, postnatal smoking exposure, and length z-score at 2 years. With robust standard errors.

For all motor domains/items except, again, the fine motor domain, there was a trend towards a positive relationship with the duration of exclusive/predominant breastfeeding. In a dose-dependent fashion, children exposed for 7 months or more had systematically higher scores compared to children that had minimal or no exclusive breastfeeding (Figs. 8 and 9). This dose-effect relationship was less clear for the visual tests and “Drinks from a cup”, an item requiring finer motor development and hand-eye coordination (Fig. 9).

Along the same lines, starting solid/semi-solid foods at 6 months or later increased the scores for these neurological markers (again, except for the fine motor domain); children with weaning delayed as late as more than 7 months had, in a dose-dependent pattern, the highest scores including for the gross motor items ‘runs’ and ‘climbs upstairs’ (Figs. 8 and 9). The effect on visual acuity followed the same pattern of positive association with the age at weaning, but limited or no effect was observed for contrast sensitivity (Fig. 8).

**Figure 10:**
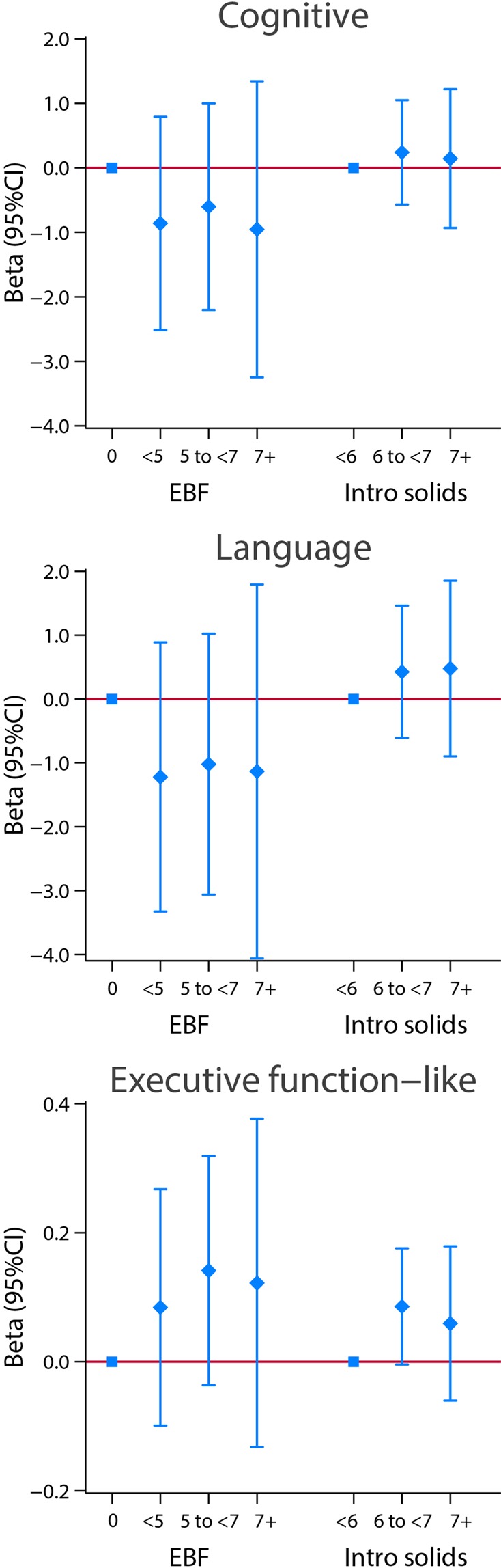
Association between categories of length of exclusive breastfeeding (EBF) and weaning age (introduction of solids), in months, and cognitive and language INTER-NDA domains at 2 years of age. Results from linear regression models adjusted for age at assessment, sex, gestational age at birth, birth weight, birth length, NICU stay, maternal education, maternal age, fetal head circumference z-score, postnatal smoking exposure, and length z-score at 2 years. With robust standard errors.

**Figure 11:**
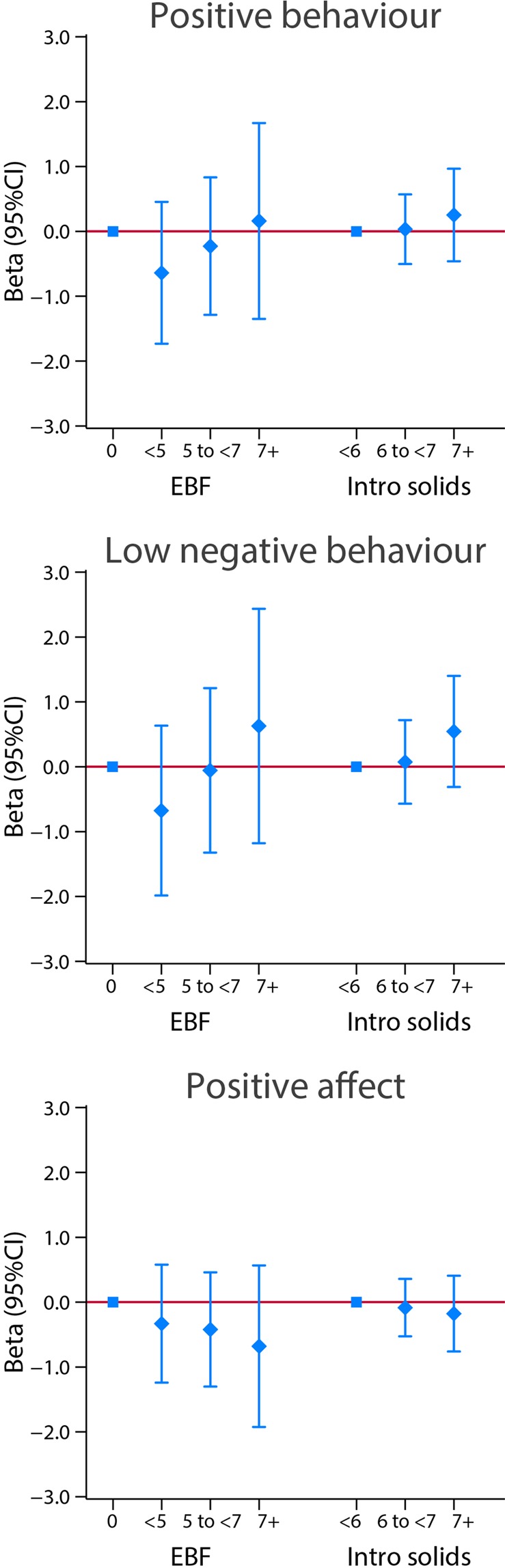
Association between categories of length of exclusive breastfeeding (EBF) and weaning age (introduction of solids), in months, and behavioural domains at 2 years of age. Results from linear regression models adjusted for age at assessment, sex, gestational age at birth, birth weight, birth length, NICU stay, maternal education, maternal age, fetal head circumference z-score, postnatal smoking exposure, and length z-score at 2 years. With robust standard errors.

Figures 10 and 11 present results using the same analytical strategy for the same two markers of feeding practices as categorical independent variables but focused here on the cognitive, language, positive and low negative behaviour domains and an “executive function-like” INTER-NDA item as the dependent continuous variables, all expressed as regression coefficients (95% CI), adjusted for the same variables as above. There were no clear patterns of association in these domains, except perhaps in the “executive function-like” item where again there was a positive association with exclusive breastfeeding and the age at weaning, although the adjusted 95% CI were wide and the regression coefficient seems not to be linear for the last stratum (Fig. 10). Similarly, no clear pattern was observed for positive and low negative behaviour, as well as for positive affect (Fig. 11).

**Figure 12:**
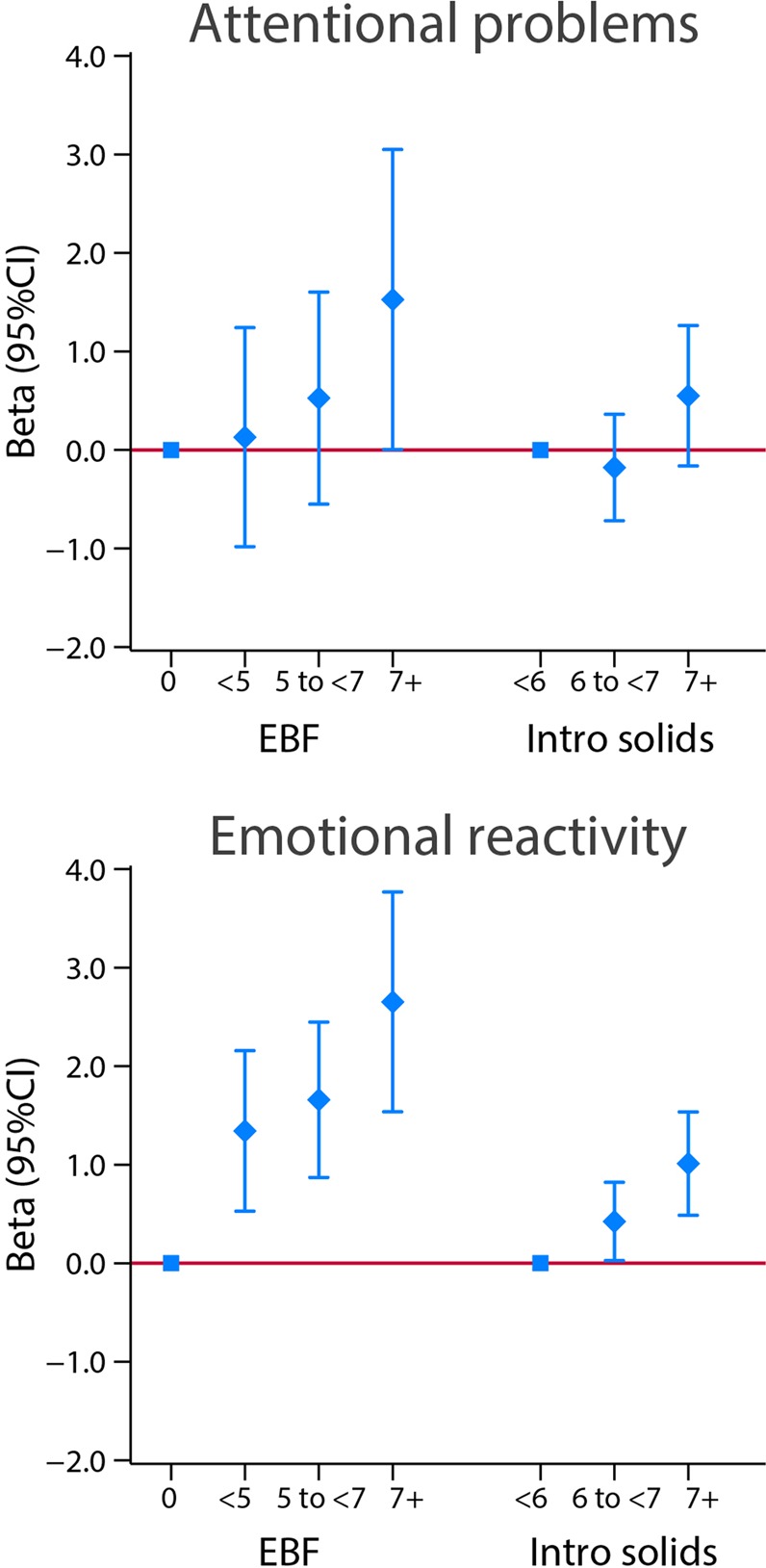
Association between categories of length of exclusive breastfeeding (EBF) and weaning age (introduction of solids), in months, and attentional “problems” and “emotional reactivity” domains scores at 2 years of age. Attentional “problems” reflects the child’s scores on the attentional problems subscale of the preschool version of the Child Behavior Checklist (CBCL). In this sample, none of the children scored above the clinical or borderline clinical thresholds for attentional problems on the CBCL; therefore, the scores reflect the variation within the normal range. The central dots represent the change in the neurodevelopmental score (with vertical lines representing 95% CIs) for each unit increase in the continuous exposure. Results from linear regression models adjusted for age at assessment, sex, gestational age at birth, birth weight, birth length, NICU stay, maternal education, maternal age, fetal head circumference z-score, postnatal smoking exposure, and length z-score at 2 years. With robust standard errors.

Conversely, there was a strong and consistent trend in exclusive/predominant breastfeeding from birth to 5–7 months and 7 months or more towards higher scores for “emotional reactivity”. Age at weaning showed the same patterns of positive association (Fig. 12). There was a positive association between exclusive breastfeeding for 7 months or more and attentional “problems” for exclusive breastfeeding and age at weaning. The results described above were identical after excluding the 29 children with clinical or borderline clinical CBCL scores.

### Association between duration of exclusive/predominant breastfeeding and age at weaning and neurodevelopmental scores according to levels of possible effect modifiers

We explored possible effect modification associated with the pre-specified variables only for neurodevelopmental domains/items that were consistently associated with months of exclusive/predominant breastfeeding and age at weaning: gross motor domain, runs and climbs upstairs. Early standing alone and early walking alone were consistently associated with these two feeding practices but the skills required are covered already by the three items selected above. An exploratory stratified analysis was also conducted with the attentional “problems” and “emotional reactivity” scores because of the consistent trends observed.

**Figure 13:**
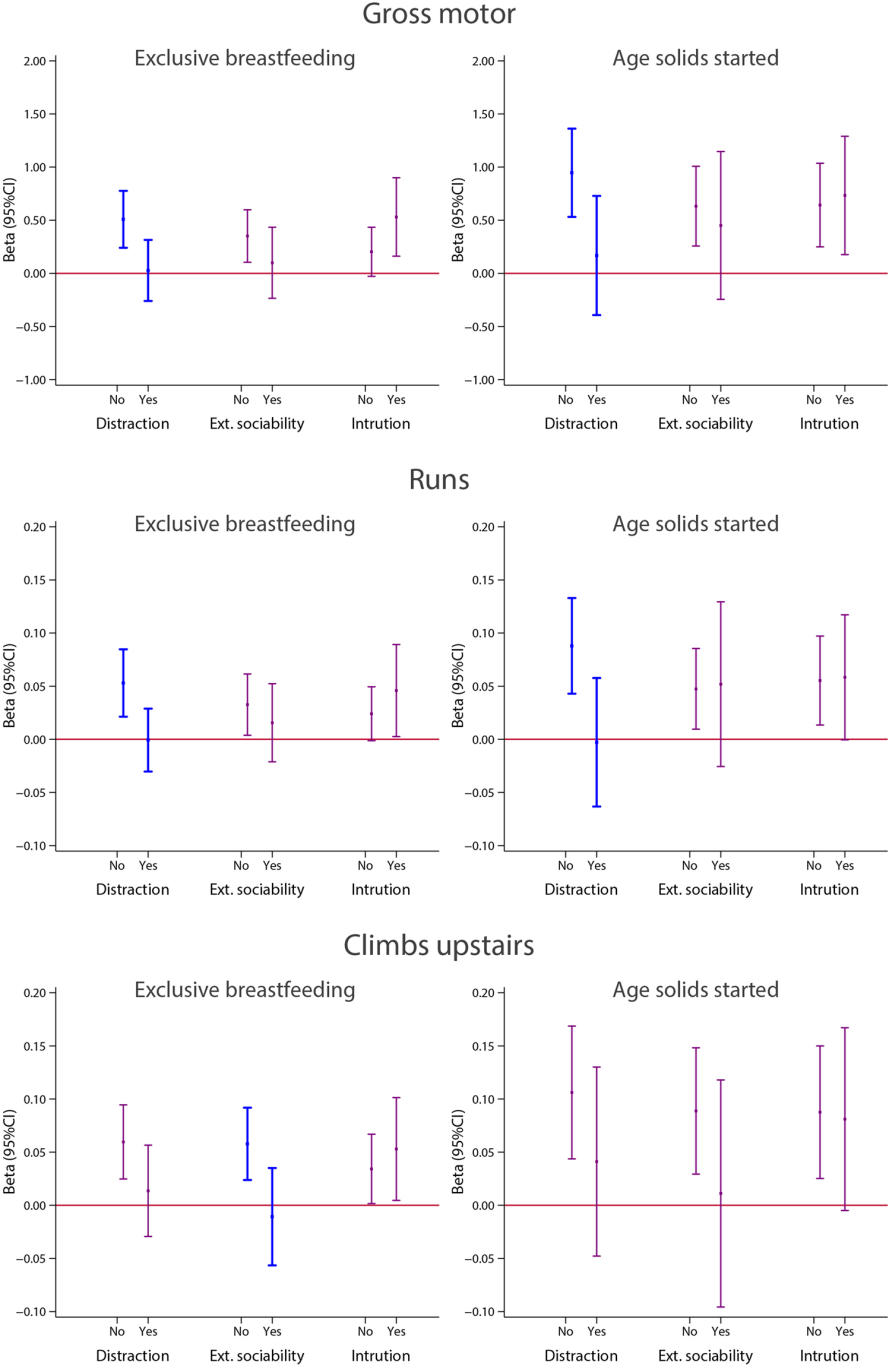
Stratified analyses for the gross motor INTER-NDA domain and two WHO Gross motor milestones items, (running and climbing upstairs), according to three potential effect modifiers: Distraction index (No = <3 distracting factors; Yes = ≥3 distracting factors, (distracting factors were defined as (a) the mother being pregnant, (b) the mother working outside the home and (c) the mother not being the main person feeding the child)); external sociability (No = child has never attended nursery; Yes = child has attended nursery); competition/intrusion (No = first child; Yes = multiparous mother). Interactions were tested using Wald tests, with p = 0.010 gross motor/distraction index; p = 0.027 gross motor/distraction index; p = 0.016 “runs”/distraction index; p = 0.017 “runs”/distraction index and p = 0.018 climbs upstairs/ external sociability. Statistically significant interactions are shown in blue. Models were adjusted for age at assessment, sex, gestational age at birth, birth weight, birth length, NICU stay, maternal education, maternal age, fetal head circumference z-score, postnatal smoking exposure, and length z-score at 2 years. With robust standard errors.

**Figure 14:**
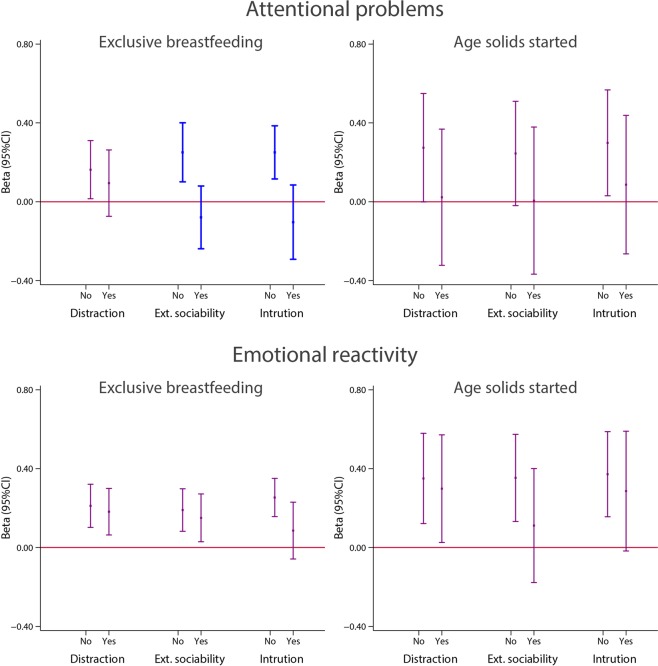
Stratified analyses for the “attentional problems” and “emotional reactivity” domains scores at 2 years of age according to three potential effect modifiers: Distraction index (No = <3 distracting factors; Yes = ≥3 distracting factors, distracting factors were defined as (a) the mother being pregnant, (b) the mother working outside the home, and (c) the mother not being the main person feeding the child)); external sociability (No = child has never attended nursery; Yes = child has attended nursery); competition/intrusion (No = first child; Yes = multiparous mother). Interactions were tested using Wald tests, with p = 0.003 attentional score/external sociability and being first child. Statistically significant interactions are shown in blue. “Attentional problems” reflects the child’s scores on the attentional problems subscale of the preschool version of the Child Behavior Checklist (CBCL). In this sample, none of the children scored above the clinical or borderline clinical thresholds for attentional problems on the CBCL; therefore, the scores reflect the variation within the normal range. The central dots represent the change in the neurodevelopmental score (with vertical lines representing 95% CIs) for each unit increase in the continuous exposure. Models were adjusted for age at assessment, sex, gestational age at birth, birth weight, birth length, NICU stay, maternal education, maternal age, fetal head circumference z-score, postnatal smoking exposure, and length z-score at 2 years. With robust standard errors.

Among the five possible effect modifiers, we did not observe any trend or association with “Gang of mothers” (care is shared with other people in addition to the mother) and “Age of the infant at the time the mother returned to work outside home”; hence, Figs. 13 and 14 only present results for the other three factors.

Figure 13 presents, as in Figs. 3 and 7, the relationship between duration of exclusive/predominant breastfeeding and age at weaning (both as continuous variables, in months) and the neurodevelopmental scores for the gross motor domain, runs and climbs upstairs items, as continuous variables according to two strata of the three possible effect modifiers. All analyses were adjusted by the same set of confounding variables as before.

For exclusive breastfeeding, there was evidence of effect modification by the “distraction” variable for the INTER-NDA gross motor domain (Wald test for interaction p = 0.016) and the WHO runs item (Wald test for interaction p = 0.016). In both cases, the positive association between longer exclusive breastfeeding and higher scores of the neurodevelopmental domain was observed for children of mothers with a low distracting index. There was a similar pattern for the positive association between exclusive breastfeeding and climbing upstairs (Wald test for interaction p = 0.018), which was observed in those children who did not attend nursery but not in those who attended nursery/preschool (Fig. 13; left hand charts).

For the age at starting solid/semi-solid foods, there were statistically significant interactions with the distraction score for the gross motor INTER-NDA domain (Wald test for interaction p = 0.027) and for the item runs (p = 0.017). Although not reaching nominal significance, a similar trend was observed for the outcomes runs and climbs upstairs if the child did not attend nursery and if the mother had a low distractions score (Fig. 13; right hand charts).

Figure 14 presents the independent relationship between duration of exclusive/predominant breastfeeding and age at weaning (both as continuous variables in months) and the scores for attentional “problems” and “emotional reactivity” (also as continuous variables), according to the same three possible effect modifiers. All analyses were adjusted by the same set of confounding variables previously considered.

There was consistent effect modification of the association between duration of exclusive/predominant breastfeeding and the attentional “problems” score according to the competition/intrusion and the external sociability factors. The association between longer exclusive breastfeeding and higher values of the attentional “problems” score was observed in the group of children who had never attended nursery and for those who were the first child (Wald test for interaction p = 0.003 for both); a similar trend was present if the mother was not working outside the home (data not shown in Fig. 14).

For the age at weaning analysis, there was again a very consistent trend toward a positive effect on the attentional gradient scores among children with high maternal closeness (Fig. 14; upper charts). The same analyses were conducted for the “emotional reactivity” outcome: duration of exclusive/predominant breastfeeding and age at starting solid/semi-solid foods were positively associated with higher scores of “emotional reactivity” regardless of the level of the stratified variables (Fig. 14; lower charts).

## Discussion

We have meticulously and prospectively studied a healthy, well-nourished, contemporary cohort of children from five diverse urban areas, free of major socio-economic constraints, whose growth and development were monitored from early fetal life to 2 years of age. We have demonstrated that measures of breastfeeding intensity and duration were positively associated with higher scores of indicators for gross motor, vision and neurological functions suggesting development of autonomy, such as running and climbing upstairs alone.

This pattern of development in healthy, well-nourished children seemingly requires a minimum critical period of exclusive/predominant breastfeeding from birth to 5–7 months of age, accompanied by a corresponding delay in weaning until after 6 months of age in keeping with WHO and UNICEF recommendations^22^. These effects follow a dose-effect relationship and are independent of a comprehensive set of confounding factors. Moreover, it appears that the magnitude of these effects is modified by variables indicative of the mother’s active and continual closeness or proximity during early infancy, which suggests that maternal-infant contact has benefits aside from those directly associated with infant nutrition.

Conversely, we did not observe any consistent relationship between breastfeeding indicators and markers of overall cognitive, language, behaviour, positive affect and fine motor development at 2 years of age. There was also no association with the fine motor domain, which comprises a range of items in the INTER-NDA, the skills for which are typically achieved between 22 and 26 months of age. It is possible that an effect on these more complex skills may become evident later, perhaps during mid-childhood or at school entry^23,24^. In general, fine motor skills in this age window should not simply be considered independent milestones; rather they provide the foundation for achieving more complex objectives beyond 2 years of age^25^.

We observed either no effect or a negative association between total time exposed to formula feeding and neurodevelopmental scores in this healthy, well-nourished population. This finding confirms what is already known about the advantages of breast over formula feeding and challenges classical theories about infants not expressing a preference for one type of milk over another, as long as their hunger is being satiated^26^.

Reassuringly, we have confirmed that breastfeeding is associated with a lower risk of morbidity, particularly infectious/allergic related diagnoses, as well as overall lower BMI z-scores based on the WHO Child Growth Standards^14^. We believe it is of great public health importance that these beneficial effects were observed in healthy, well-nourished populations across diverse geographical locations.

We appear, therefore, to be describing a maturational sequence in which a degree of maturity in gross motor, neurological and visual skills is required as the foundation for the development of further, more complex and advanced skills. Our results also indicate a strong “dose-dependent” increase of the maternally reported “emotional reactivity” (all within normal values of the scale)^21^, according to the duration of exclusive/predominant breastfeeding and timing of weaning, after adjusting for a comprehensive set of possible confounding variables.

Our study has unique features and limitations. The INTERGROWTH-21^st^ Project is unique because a large cohort of children with adequate health and nutrition, at low risk of social, educational and economic disadvantage, were monitored prospectively from early fetal life to 2 years of age, under well-controlled and standardised conditions^11,12^. This allowed us to explore early neurodevelopment and associated behaviours in a sample of children growing up in environments that were low risk for detrimental effects on the attainment of developmental skills.

This amount of methodological rigour and standardisation, which included assessment of growth and morbidity, has seldom been achieved for such a large, cross-national sample in studies of child development starting in early fetal life^27^. There have been retrospective accounts of adults, or adults describing their children’s experiences, usually involving small samples albeit with reports over many years. Other studies, although prospective and very detailed (even daily reports), have included few children or children with specific socio-cultural-psychological conditions or pathologies^25,28,29^. Moreover, the sampling frame most has not been international. The possibility of observational and reporting bias in many of these studies cannot be ignored because there was limited masking during the collection of outcome measures.

Secondly, we used a number of neurodevelopmental domains and specific items that provided multiple and varied sources of data. In addition, we tried to answer a set of questions without adhering to any particular philosophical, psychoanalytical or scientific school of thought to enable us to explore a wide range of possible interpretations in an unbiased, integrated manner.

As a result of the strategy described above, the study had some limitations. The number of items per domain used to evaluate neurodevelopment and associated behaviours varied, and some scores were based on only a few items. This is an inevitable consequence of using reduced psychometrics (which are required in large, free-living human samples) and aiming to understand different developmental areas rather than summary quotients. Hence, we acknowledge the exploratory nature of such analyses. Nevertheless, we consider that our approach of exploring different domains in the same sample is key to understanding such complex processes which have traditionally been conceptualised independently, e.g. cognition separately from affective domains, and even separately from language.

Another limitation, which is common in complex observational clinical research, is the use of multiple statistical testing (regression coefficients (95% CI) or OR (95% CI)) without pre-specified effect sizes or even the direction of the effect^30^. We have used formal statistical testing, implicit in the 95% CI, as one of several elements to interpret the results, but mostly focused on trends of the effect and the association in a “dose-effect” fashion rather than a single response to a possible effect. Furthermore, the number of observed associations is higher that would be expected by chance alone.

Two recent systematic reviews have discussed issues related to residual confounding in this field, including studies with a strong multivariable analysis component^27,31^. Hence, we have adjusted by a comprehensive set of possible universal and topic-specific confounding variables, using multilevel, linear regression analysis, complemented by logistic regression. The very close pattern of the results and shape of the effect obtained using these two analytical strategies are reassuring; it is unlikely that the observed systematic trends would change with further adjustments.

We have also clearly separated the statistical treatment of confounding variables (by controlling for them) from the exploration of effect modification that we have evaluated in stratified analyses of variables or indices selected *a priori*. Finally, we consider the estimations rather conservative, because the number of children without exclusive breastfeeding (the reference category) is relatively small, which increases the standard error, widening the confidence intervals.

We have documented breastfeeding only in quantitative terms (duration of the exposure and intensity) without providing any exteroceptive information about mother-child dyad interactions, visual interchanges, touching, skin contact or bonding, all of which are almost certainly as important biologically as nutritional, developmental and immunological factors in these healthy populations. It was impractical to collect such detailed, personal data in a large, multi-country cohort study such as ours. Nevertheless, we made two assumptions: (1) the degree of visual/physical interaction is proportional to the intensity of breastfeeding and maternal closeness and (2) there was no systematic bias because breastfeeding recommendations and support were promoted by the local institutions unrelated to the nature of the hypotheses being tested in the present analysis. At the same time, we recognise that: (1) in some women, short breastfeeding periods may have been associated with intense mother-child interactions and (2) some infants might have been fed expressed breast milk by a caregiver other than their mother, biasing the intensity of maternal closeness. However, we were unable to document such events.

The study had a relatively short postnatal follow-up period of 2 years and it would have been better to have data up to at least school age. However, there are many obvious practical, economic and logistical factors affecting the selection of a later milestone in the follow-up of large multicentre cohorts.

Nevertheless, it is generally agreed that 2 years of age represents the end of a fundamental period of human development, the “sensorimotor period”^25^ or “situational intelligence”^32^ or “the physiological beginning”^33^, or the end of the “trust-mistrust” and the beginning of the “autonomy-shame” stages^34^, which is followed in the third year of life by the initiation of mental representation, initial thoughts and active socialisation. Therefore, in our opinion, evaluation at 2 years of age provides an acceptable compromise between field logistics and the desire to assess the child at as advanced a stage as possible in the developmental sequence of maturation.

It is important to emphasise that this was not a study that addressed the effects of human milk or breastfeeding on health and nutrition. There is a very large literature on this subject, including systematic reviews^17,35^ and narrative discussions^36^ providing evidence about the benefits of human milk and breastfeeding with their role of nutrient/nursing^37^, particularly in less developed countries^4,16^, and it is reassuring that our results in a healthy population support those conclusions.

We have used as the primary exposure, in addition to duration and intensity of breastfeeding, the age at first introduction of solid/semisolid foods, i.e. weaning, as a point that can be objectively measured, marking the initiation of the process of separation from the “m-other”. The appropriateness of doing so is strengthened by understanding the derivation of the word weaning, which comes from the Anglo-Saxon word “wenian” meaning “to become accustomed to something different”.

These two processes are recognised by most developmental theories as important contributors to the formation of fundamental human mental structures and circuits. Importantly, it can be said that it is only during this short period of human life that conceptual issues such as initiation of “object relations”, dependency, dependence and attachment converge.

All things considered, we believe that the evidence presented here on the timing, sequence and interactions of early feeding patterns with specific human neurodevelopmental skills and associated behaviours, covers a number of previously proposed (often separately) concepts and theories, which we briefly discuss below in an attempt to integrate them empirically.

Our findings provide strong evidence that the effect of breastfeeding on early child development may be mediated by an initial selective effect on gross motor development, facilitating more active, exploratory, independent activities as reflected in items such as running or climbing upstairs alone during the first 18 months of life. According to Piaget, during this initial sensorimotor stage of cognitive development from birth to approximately 2 years of age, infants derive their understanding of the world through active adaptation to their environment^25^.

By the 8-month stage, the child has acquired “object permanence”. This is the understanding that objects exist independent of one’s own actions, a step essential for the next stage of development - acquiring the capacity to produce mental “imagery”. These concepts are compatible with our observations of association with specific sensorimotor functions, without overall effects on cognition or behaviour, perhaps because the latter require mental capacities that only become available after 2 years of age.

Similarly, we observed a positive effect of weaning after 6–7 months of age on visual acuity and of exclusive breastfeeding on visual contrast (Fig. 8). In other words, breastfeeding is important during the stage at which vision and actions begin to be coordinated, e.g. grasping or prehension^25^, and when smiling is focused mostly on seeing the mother or friends as a love object^33^.

We believe our data tend to support Piaget’s theory that a degree of sensorimotor development is required well before abstract intelligence can manifest itself: in short, that “cognition evolves after motor action”^38^. Moreover, evolutionary biology acknowledges that, for most mammals, physical skills and vision are among the first neurodevelopmental skills to mature and develop in a predictable way during the early years of life^39^.

It is not surprising that our empirical data are supportive given the similarities in the two conceptual frameworks. The INTERGROWTH-21^st^ Project was conceived to conduct mostly normative research on early human growth and development at population level with the aim of producing international standards for use worldwide^10^. Piaget’s work focused on “normative science” or the presentation of universal laws of development from birth to adolescence, also at population level^25^. Hence, both provide evidence to support universal laws governing how humans grow and develop.

Human milk components are likely to be contributing to these maturational processes: for example, carotenoids and luteins accumulate in the infant retina and brain, and have a beneficial effect on visual function^40,41^. In infant rhesus macaques, breastfeeding was better than formula feeding at promoting the maturation of the corpus callosum and cerebral cortical gray matter, measured at 2, 4 and 6 months of age^42^. A similar longitudinal neuroimaging study in children from 3 months to 9 years of age showed significantly improved overall myelination accompanied by increased general, verbal, and non-verbal cognitive abilities in those who were breastfed compared to children who were exclusively formula fed^43^.

The lack of association of exclusive/predominant breastfeeding and age at weaning with cognition, behaviour, affection and language can have several explanations. First, these domains were assessed at 2 years of age, i.e. in the very initial stages of structure formation when only the most basic psychological functions have developed. More advanced levels are achieved later with more profound social relationships^44^, and perhaps the amount of “intelligence” present at this age is too low to be measured with our tools.

An alternative view is that traditional psychometric tools focus mostly on quantifying “individual cognition”, i.e. the very young child is tested alone, as the “solitary child”. However, at this age, it is possible that children can perform some of the requested tasks, but they need direct help from others or supporting representations using, for example, language. Hence, we have started to test children at 2 years of age in small groups to capture social “cognitive” skills^45^, and there seems to be some interest in the field for capturing more ecologically valid skills^46^.

We have observed a consistent positive effect of late weaning similar or stronger to that of exclusive/ predominant breastfeeding, which could simply be a different manifestation of the same process, i.e. longer exposure to breastfeeding. However, previous clinical and theoretical work support the concept that the timing of weaning, as a socio-culturally dominated process, is expected to be independently related to early neurodevelopment, behaviours and long-term psychological effects.

For example, Klein described a “depressive position” after 5 months of age due to the partial break-up of the intense relationship with the mother^47^, and interruption of the rhythmic and pleasurable regularity of being fed at the breast. In 1938, Lacan described a “weaning complex (complexe du sevrage)” as a link between mental representations and the earliest sets of imagines of the mother/family to be registered in the mind^48^.

Perhaps our most intriguing finding is the systematic positive association between markers of breastfeeding and age at weaning, and higher scores in the “emotional reactivity” and attentional “problems” scores based on maternal perception. The interpretation of these results requires an understanding of the CBCL scale as applied in our sample at 2 years of age (a special developmental period within the age range of this scale), as well as how these results match the theories available.

The CBCL^21^ obtains data from the mother (i.e. there is no direct observation of the child) to detect a wide range of problem behaviours requiring further evaluation between 1½ to 5 years of age. The instrument, as expected, has inherent maternal recall biases and in-built preconceptions relating to present-day, westernised, social dynamics and small family sizes.

We studied low-risk children with mean/median scores, graded using the official CBCL scoring system^21^, that were well within normal range; in a sensitivity analysis, we excluded the few children with high, borderline clinical scores without any effect on the overall values. Hence, our results should be interpreted as a variation of non-clinical attentional “problems” within the normal range, rather than selection of abnormal children. In this context, our results show that the longer the exclusive/predominant breastfeeding and later the weaning, the more “active”, “rebel”, “refuse to follow indications”, “does not pay attention” children are perceived to be at 2 years of age by 21^st^ century, low parity and educated mothers.

How do these observations relate to developmental theories? By 2 years of age, it has been suggested that children enter a period of “stubbornness” or rebellion against their mother/caregiver by for example, walking away or having tantrums: the “terrible twos”^33^. This could be a consequence of the multiple prohibitions and controls that are imposed on active and dynamic children. To transit through this period with confidence, the child must attain a degree of neuromotor maturation that gives both physical and emotional *independence*, including the capacity to express frustration, Spitz’s classic “no” period^33,49^.

To initiate such non-verbal communication to reduce *dependence* on the mother, it is necessary to have: (a) the physical capacity and security to be at a distance from her, taking control over physical skills; in our data, running, climbing upstairs and gross motor development in general and (b) the “confidence” to express frustration and aggressiveness, and to refuse orders; in our data, higher “emotional reactivity” and attentional “problems”. It can be fully achieved if the child knows that their basic *dependency* and affectional ties will be satisfied, i.e. “basic trust” is ensured^50,51^. A recently reported positive association between duration of breastfeeding and secured, organised maternal attachment by 2 years of age, supports this interpretation^52^.

We explored five effect modifiers we considered to be proxy indicators of the mother’s closeness to the child. Our results showed a consistent pattern for three indicators of closeness (describing emotional and physical interaction): the positive association between exclusive breastfeeding and age of weaning on gross motor functions was concentrated in those mothers who seem to have been closer to, and less distracted from, their infant. Although we lacked details about the nature of their relationship, mothers who breastfeed tend to interact more physically and visually with, and respond more actively towards, their infant, and longer breastfeeding periods increase maternal sensitivity when interacting during childhood^52^.

For the avoidance of doubt, we are not promoting a specific type of interaction nor the need for the mother to be constantly present throughout infancy, because care is often now provided by a variety of family members and caregivers. Rather, our results support the concept that, during this multiphasic, fundamental period of human development in the first 6 to 8 months, there is a confluence of inter-connected environmental factors and mental processes that, by the time of the infant’s second birthday, have different maturational influences (as described here), on their psychological make-up.

According to some theories, these factors include the duration of breastfeeding and timing of weaning; sibling rivalry; the real or symbolic mirror stage^32,53^, a “depressive position” with the introduction of solids^54^; the identification of the “other” and Spitz’s executive stage, i.e. smiling to all^33^. Whether or not such expressions will re-emerge later in life as real or imaginary, or reflect intelligence, sociability or clinically relevant phenomena was not the objective of our normative project.

We fully recognise that developmental experts with different conceptual frameworks may not interpret our results in the same way. We more than welcome other contributions and hope they will be used for the design of future studies to explore alternative interpretations, avoiding the limitations of our project.

Ultimately, we hope that the evidence presented here, supporting the overlapping of developmental theories that are closer than generally accepted, contributes to a unified understanding of early human development, which will lead to more effective clinical treatments and preventive interventions. We also encourage the evaluation of complex exposures and interventions in the field of early child development with the use of domain specific outcomes rather than summary psychometrics, in the context of large, long-term, multi-national follow-up studies or randomised clinical trials.

## Materials and Methods

INTERGROWTH-21^st^ was a large, multicentre, population-based, research project conducted between 2009 and 2016, in eight delimited urban areas across five continents. The primary aim was to study growth, health, nutrition and development from early pregnancy to 2 years of age in mothers and children with adequate health, nutritional, environmental and socio-economic conditions at both individual and population levels^10^.

FGLS, one of the main components of the INTERGROWTH-21^st^ Project, included pregnant women from these eight populations, who met the individual entry criteria^13,55–58^. Children from the cohort of mothers enrolled during pregnancy in FGLS were followed up to 2 years of age (the Infant Follow-up Study (IFS)), and evaluated for growth, nutrition, health and developmental outcomes (the WHO gross motor milestones)^11,59^.

All participants contributed data towards the construction of the international INTERGROWTH-21^st^ Fetal Growth and Preterm Postnatal Growth Standards^13,58^. At the 2 year IFS visit, children completed a comprehensive neurodevelopment assessment in five of the eight original sites: the cities of Pelotas (Brazil); Turin (Italy); Oxford (UK); the central area of Nagpur (India) and the Parklands suburb of Nairobi (Kenya), using a set of tools specifically developed or selected for this purpose^60^. The sites in China, Oman and the USA did not participate in this phase because of local logistical and administrative reasons, all unrelated to the nature of the questions explored in the follow-up study.

Across all study sites, we implemented standardised clinical care and infant feeding practices based on protocols developed by the INTERGROWTH-21^st^ Neonatal Group (www.intergrowth21.org.uk). Exclusive breastfeeding up to 6 months was promoted^61^. During pregnancy, at birth and at 1 and 2 years of age, standardised information was obtained on health, anthropometric measures, severe morbidities, duration of breastfeeding, timing of the introduction of solid and semi-solid foods, feeding practices and food intake (www.intergrowth21.org.uk).

We excluded children with severe morbidities up to 2 years of age, such as tuberculosis, hepatitis, HIV/AIDS, malaria, hearing problems, neurometabolic conditions, epilepsy, meningitis, seizures, cerebral palsy, cardiovascular problems, cystic fibrosis, blindness, haemolytic disorders and any malignancy. The baseline characteristics of the full cohort and follow-up methodology have recently been published^11^, as well those of the developmental sample^12^.

### Neurodevelopment outcomes

The INTERGROWTH-21^st^ Neurodevelopment Assessment (INTER-NDA) is a brief, objective, psychometric tool, measuring multiple dimensions of early child development, targeted at children aged 22–30 months^60^. We designed the tool specifically for implementation by non-specialists across international settings^62^. It consists of 37 items measuring cognition, expressive and receptive language, fine and gross motor skills, and positive and negative behaviour using a combination of directly administered, concurrently observed and caregiver reported items^60^. In addition, we also administered the attentional problems and emotional reactivity sub-scales of the CBCL to our study population^24^.

The INTER-NDA shows good to moderate agreement with the Bayley Scales of Infant Development III edition^62^, which is considered the gold-standard child development assessment at individual level for screening and monitoring purposes^63^. It also has good levels of inter-rater (k  =  0.70; 95% CI: 0.47–0.88) and test/re-test reliability (k = 0.79; 95%CI: 0.48–0.96)^60^.

INTER-NDA data were collected using a tablet-based system developed specifically for IFS, with an incorporated operation manual as well as visual cues, examples and fully integrated quality checks^60^. Staff administering the assessments were aware of the study’s general principles but not the specific hypotheses being tested. Data were uploaded to an encrypted, cloud-based server as soon as each assessment ended.

The INTER-NDA’s negative behaviour domain describes negative aspects of a child’s behaviour, observed during the assessment, beyond what is expected for the child’s age. It includes ratings on distractibility items (poor attention to tasks, easily distractible, leaves tasks incomplete) and negative affect items (excessive tantrums, fussing, pouting, whining, crying and aggressive outbursts). For these analyses, we reversed negative behaviour scores to allow them to be expressed in a positive format, i.e. a higher ‘low negative’ behaviour score is more favourable.

Individual children’s performance on the CBCL’s attentional “problems” and emotional reactivity subscales^21^ were estimated as the mean value for individual scores from the raw data and as CBCL centiles to identify children with scores above the clinical range on the CBCL, i.e. greater than 97^th^ centile of their transformed distribution.

The age of achievement of the gross motor development milestones “standing alone” and “walking alone”, as defined by WHO^59,64^, were evaluated for their association with feeding practices. There were some discrepancies between the reports at 1- and 2-year follow-up visits for 19 children out of 1292 for “standing alone” and 18 children out of 1296 for “walking alone”; in these cases, the 1-year information was preferred.

The two gross motor development milestones were modified to correspond to an “earlier age for standing” and an “earlier age for walking”. The new variables represent the difference between 26 months and the age at which the child stood or walked alone (i.e. the larger the value, the earlier the child stood or walked on their own, in exact months).

Vision was assessed using the Cardiff Visual Acuity and Contrast Sensitivity tests for binocular vision^65^.

We identified *a priori*, three items for collection by direct observation at the time of the assessment, as indicators of the child’s autonomy i.e. independence from the mother: runs alone, climbs upstairs and drinks spontaneously from a cup deposited on the nursery table in front of the child, without any inducement from the examiner or mother. These were analysed as separate items.

INTER-NDA items were scored on a four-point scale (range 1 to 4) and behavioural items were scored on a three-point scale (range 1 to 3 for observed behaviour and 0 to 2 for caregiver reported questionnaire items). The mean domain scores were multiplied by a factor of 10 to make it easier to interpret the results. Items related to the child’s independence from the mother and the INTER-NDA’s “executive-function like” item were analysed using their original scale.

### Feeding practices

The main independent variables were those describing feeding practices, i.e. breastfeeding, formula feeding, introduction of solid/semi-solid foods, and number of feeds per day as reported at the 1-year visit. These exposures were evaluated as continuous variables, e.g. number of months of exclusive/predominant/any breastfeeding or age in months at which solid/semi-solid foods (weaning) were introduced, following working definitions recommended by WHO^66^.

1. Duration of exclusive/predominant breastfeeding (months): total time the child was fed only breast milk or breast milk as the predominant source of nourishment allowing for certain liquids (water and water-based drinks, fruit juice), ritual fluids and drops or syrups (vitamins, minerals, medicines).
2. Duration of any breastfeeding (months): total time the child was exposed to breast milk either as exclusive breastfeeding or breast milk, plus any food or liquid including non-human milk and formula.
3. Duration of formula use (months): total time the child was exposed to formula by the 2-year visit, either as exclusive formula or in combination with other feeding practices.
4. Number of breast feeds per day: as reported at the 1-year visit.
5. Age (months) at which solid/semi-solid foods were introduced: age of the child when solid/semi-solid foods/family or adult foods were introduced for the first time as reported by the mother^67^.

There were children who received milks other than formula as part of bottle-feeding practices; we used these data to refine the duration of exclusive/predominant breastfeeding variable.

As the age at initiation of formula feeding could vary for children with the same duration of total time of exposure, analyses were adjusted by the age at which formula feeding started (linear regression models) or by indicators of the timing of exposure at critical ages: hospital discharge, 3, 6, and 9 completed months (logistic regression models). The latter strategy was chosen given that adjustment for the age of the start of formula feeding produced multi-collinearity in the multivariable models.

Data for duration of exclusive/predominant breastfeeding were transformed into categorical variables to study the patterns of association between the timing of feeding and neurodevelopmental scores. To determine the cut-off points for this categorisation, we first conducted a review of the developmental periods traditionally proposed as key for the acquisition of psychological milestones^25,33,34,44,68–70^.

We then established cut-off points for exclusive/predominant breastfeeding and age at weaning from birth to the postnatal ages less than 5 months, less than 7 months, less than 13 months and less than 18 months of age. Regardless of the terminology used, it is generally accepted that fundamental neurodevelopmental processes occur within these approximate time-windows. Thus, we expected to capture, for example, the effect on early child development of being exclusively breastfed from birth to 5 months as compared to a further 2 months of exclusive/predominant breastfeeding, i.e. from birth to less than 7 months of age.

### Statistical analysis

Linear regression analyses (crude and adjusted) were conducted to explore associations separately for each feeding exposure as a continuous or categorical (independent) variable against each neurodevelopmental domain as a continuous (dependent) variable. Robust standard errors were estimated in all linear association models.

For adjustment, the following variables were taken into account when assessing the association between feeding practices and neurodevelopment: fetal head circumference z-score between 25 and 30 weeks’ gestation based on the INTERGROWTH-21^st^ Fetal Growth Standards, sex (girls as the baseline group), gestational age at birth (in exact weeks, corroborated by ultrasound assessment at less than 14 weeks’ gestation), birth weight (kg), birth length (cm), NICU stay (yes or no), age of the child at the time of the neurodevelopmental assessment (in exact months). Height z-score at 2 years of age based on the WHO Child Growth Standards^14^ was not considered a confounder but rather a possible mediator; nevertheless, it was included in the models to explore the independent, non-nutritional effect of early feeding exposures. Finally, we also evaluated the possible confounding effects of maternal age, maternal education and postnatal environmental smoking exposure^71,72^.

The relationship between study site and infant neurodevelopment has been already studied in this population, showing the two were only marginally associated^12^. Furthermore, its inclusion in the regression models could lead to over adjustment particularly given the extensive adjustment for individual characteristics already in place^73^.

We selected neurodevelopmental domains for which a consistent association or trend was observed with breastfeeding exposures, and further explored possible effect modification by indicators suggested in the literature to be related to the mother-child dyad. Hence, we repeated analyses for gross motor, runs, climbs upstairs, visual acuity, attentional score and emotional reactivity as dependent variables, and exclusive breastfeeding and age at starting solid/semi-solid foods as independent variables, all as continuous scores, stratified by the following variables considered *a priori* as potential effect modifiers.

We constructed these indices/variables because we considered them proxy indicators of maternal closeness, i.e. indicators of emotional and/or objective physical maternal-infant interaction: (1) “Distracting mother-infant relationship index”, constructed by adding up indicators for the mother: (a) being pregnant, (b) working outside the home, and (c) not being the main person feeding the child. Each indicator contributed a value of 1 for each year present, giving the score a theoretical range from 0 to 6 units. We defined categories for low distracting score (<3) versus distracting score (≥3); (2) “Gangs of mothers” effect, defined as the mother being the main person feeding the child versus another person (Gangs of mothers); (3) “External sociability”: if the infant attended a nursery school or similar facility outside the home in the first or second year of life, defined as not attending nursery versus attending nursery either year; (4) “Competition or Intrusion”: to explore the “competitive” feelings an infant might have for its mother when it realises it has siblings, i.e. “the infant is not alone”. The number of siblings was used as a proxy, defined as no siblings versus any sibling; and (5) “Age of the infant at the time the mother returned to work outside home”: stratified as a categorical variable that took the value of 0 if the mother did not work during the first 2 post-partum years; 1 if the mother went on to work when/or after the child was 6 months old, and 2 if the mother worked outside the house before the child was 6 months old.

Infant morbidity was evaluated in the second year of life by creating an unweighted score including non-severe conditions such as repeated pneumonia, urinary tract infections, glomerulonephritis, metabolic disorders, type-1 diabetes and/or ketoacidosis, i.e. any condition requiring surgery or admission to hospital.

A score related to infectious/allergic/autoimmune conditions was constructed separately, involving the following diagnoses: exanthema skin disease, repeated otitis media, repeated pneumonia, urinary tract infections, recurrent fever episodes, and gastrointestinal infections including repeated diarrhoea and persistent vomiting. In adjusted models, both morbidity scores were introduced as binary indicators given the small number of children with more than one morbid event for models including categorical exposures.

Associations between breastfeeding practices and the two morbidity indicators were assessed in logistic regression models adjusted for the same covariates used in the multivariable linear regression models.

For all analyses, Stata 15 software was used (StataCorp. 2017. Stata Statistical Software: Release 15. College Station, TX: StataCorp LLC). Data were entered locally into the specially developed, online data management system (http://medscinet.com)^74^.

The INTERGROWTH-21^st^ Project was approved by the Oxfordshire Research Ethics Committee “C” (reference: 08/H0606/139), the research ethics committees of the individual institutions and the regional health authorities where the project was implemented. Parents gave written, fully informed consent, on behalf of their children for their participation in the study. The sponsors had no role in the study design, data collection, analysis, interpretation of the data, or writing of the paper. The following authors had access to the full raw dataset: JV, ESU and SHK. The corresponding author had full access to all the data and final responsibility for submitting the paper. All methods were performed in accordance with the relevant guidelines and regulations.

## Supplementary information

Supplementary Figures 1 and 2.

## Data Availability

The authors agree to make the data available upon reasonable request.
