# Supplementary material for: Late weaning and maternal closeness, associated with advanced motor and visual maturation, reinforce autonomy in healthy, 2-year-old children
Source: Sci Rep. 2020 Mar 23;10:5251. doi: 10.1038/s41598-020-61917-z (PMC7090084; doi:10.1038/s41598-020-61917-z)
Supplement: Supplementary file 1 — Supplementary Figures 1 and 2. [file 41598_2020_61917_MOESM1_ESM.pdf]

Supplementary Material

Late weaning and maternal closeness, associated with advanced motor and visual maturation, reinforce autonomy in healthy, 2-year-old children

José Villar*[1] , Roseline Ochieng[2] , Eleonora Staines-Urias[3], Michelle Fernandes[3] , Marc Ratcliff [4,] Manorama Purwar[5], Fernando Barros[6], Bernardo Horta[7], Leila Cheikh Ismail[8], Elaine Albernaz[9], Naina Kunnawar[5], Sophie Temple[3], Francesca Giuliani[10], Tamsin Sandells[3], Maria Carvalho[2], Eric Ohuma[11] Yasmin Jaffer[12], J.Alison Noble[13], Michael Gravett[14], Ruyan Pang[15], Ann Lambert[1], Enrico Bertino[16] Paola Di Nicola[10], Aris Papageorghiou[1], Alan Stein‡[17], Zulfiqar Bhutta‡[17] & Stephen Kennedy‡[1,2]

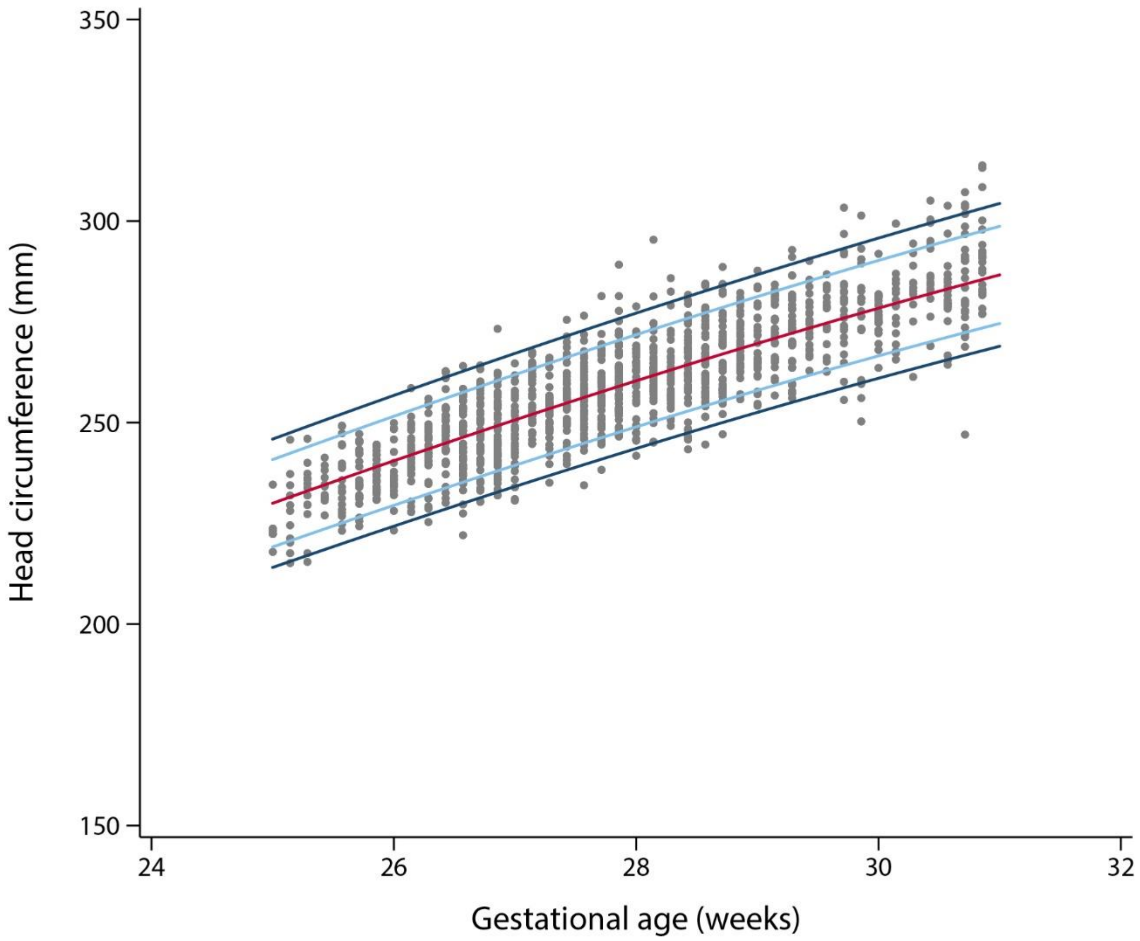

Supplementary Figure 1. Ultrasound measurements of fetal head circumference taken between 25 and 30 complete weeks of gestation for the children included in the INTERGROWTH-21st Neurodevelopment Assessment Study at 2 years of age. Superimposed are the 3rd (dark blue), 10th (light blue), 50th (red), 90th (light blue) and 97th (dark blue) Fetal Growth Longitudinal Study standard centile curves for fetal head circumference according to gestational age[13].

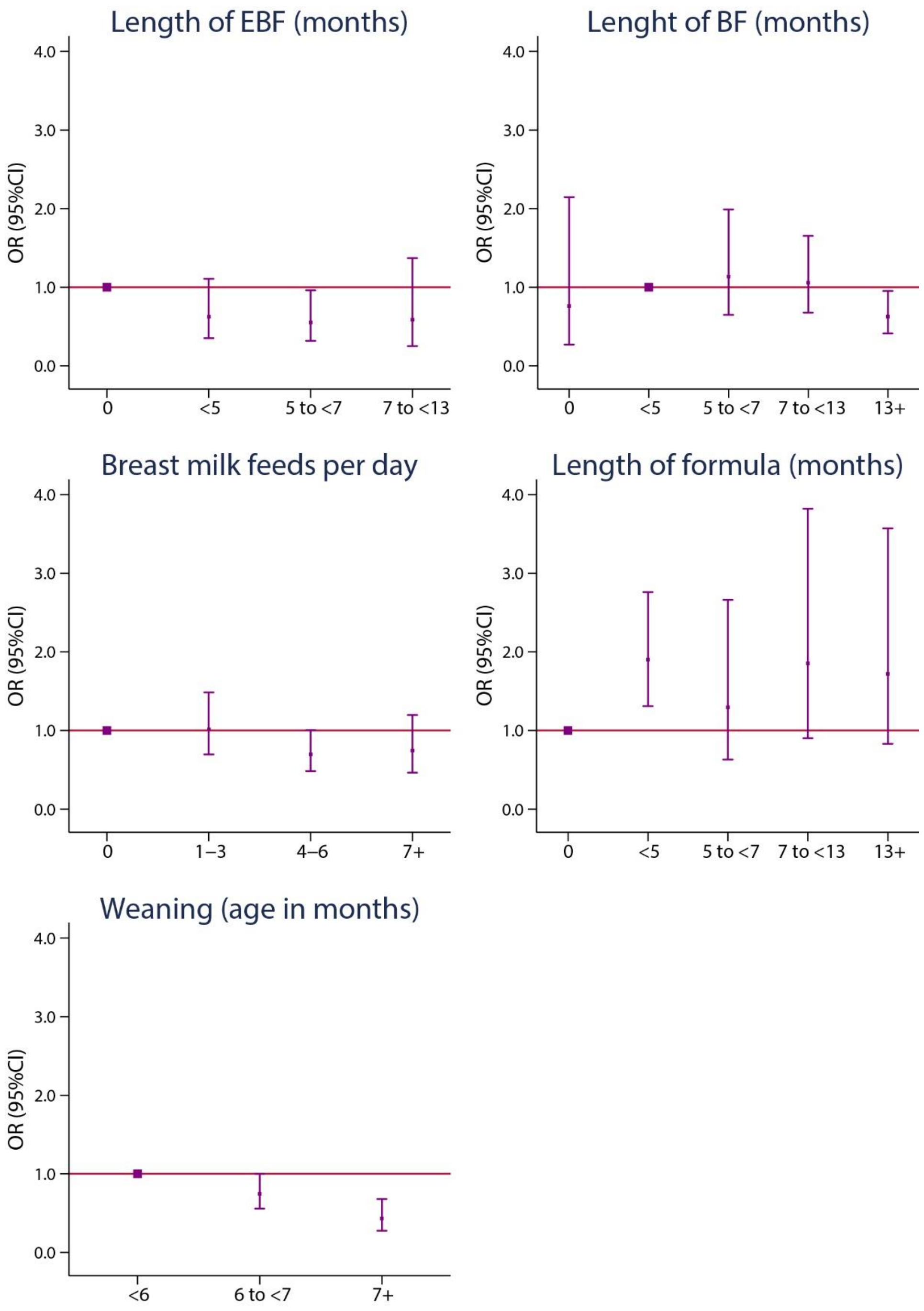

Supplementary Figure 2. Adjusted odds ratios (95% confidence intervals) for five breastfeeding exposures and infection-related morbidity rates by the second year of life. Models were adjusted for age at examination (months), sex, gestational age at birth, birth weight, birth length, NICU stay, maternal education, maternal age, fetal head circumference Z-score, postnatal smoking exposure. For formula use, we further adjusted by the use of formula during critical times (birth, 3, 6, and 9 completed months).
